# Highlight on the Mechanism of Linear Polyamidoamine Degradation in Water

**DOI:** 10.3390/polym12061376

**Published:** 2020-06-19

**Authors:** Matteo Arioli, Amedea Manfredi, Jenny Alongi, Paolo Ferruti, Elisabetta Ranucci

**Affiliations:** Dipartimento di Chimica, Università degli Studi di Milano, via C. Golgi 19, 20133 Milano, Italy; matteo.arioli@studenti.unimi.it (M.A.); amedea.manfredi@unimi.it (A.M.); jenny.alongi@unimi.it (J.A.)

**Keywords:** degradable polymers, linear polyamidoamines, degradation mechanism, retro-aza-Michael reaction

## Abstract

This paper aims at elucidating the degradation mechanism of linear polyamidoamines (PAAs) in water. PAAs are synthesized by the aza-Michael polyaddition of prim-monoamines or bis-sec-amines with bisacrylamides. Many PAAs are water-soluble and have potential for biotechnological applications and as flame-retardants. PAAs have long been known to degrade in water at pH ≥ 7, but their degradation mechanism has never been explored in detail. Filling this gap was necessary to assess the suitability of PAAs for the above applications. To this aim, a small library of nine PAAs was expressly synthesized and their degradation mechanism in aqueous solution studied by ^1^H-NMR in different conditions of pH and temperature. The main degradation mechanism was in all cases the retro-aza-Michael reaction triggered by dilution but, in some cases, hints were detected of concurrent hydrolytic degradation. Most PAAs were stable at pH 4.0; all degraded at pH 7.0 and 9.0. Initially, the degradation rate was faster at pH 9.0 than at pH 7.0, but the percent degradation after 97 days was mostly lower. In most cases, at pH 7.0 the degradation followed first order kinetics. The degradation rates mainly depended on the basicity of the amine monomers. More basic amines acted as better leaving groups.

## 1. Introduction

Linear polyamidoamines (PAAs) are the aza-Michael polyaddition products of prim-monoamines or bis-sec-amines with bisacrylamides [1,2,3]. A recent development of PAA chemistry consisting of employing natural α-amino acids as monomers has led to polyamidoamino acids (PAACs), a novel class of polymers deriving from natural α-amino acids, which maintain the α-amino acid chirality and amphoteric properties [4,5,6,7,8,9]. The controlled synthesis of PAACs has recently been reported [10]. Both PAAs and PAACs are endowed with remarkable potential for biomedical and biotechnological applications [11,12,13,14,15,16,17,18]. More recently, several PAAs and PAACs have proven to be excellent flame-retardants for cotton [19,20,21,22]. Other applications include, among others, coatings for sensors [23,24,25] and heavy metal ion sorbents [26,27,28,29]. It was soon realized that PAAs [30] and PAACs [4] slowly degrade in aqueous solution at pH ≥ 7, as confirmed by monitoring the decrease of molecular weight on time in dilute solutions [31,32,33]. 

Degradability in aqueous media is a relevant property of PAAs. Tuning PAA degradation and identifying the nature of PAA degradation products is of paramount importance for predicting their behavior in different model environments, as for instance biological fluids, aquifers and wet soil. The degradation mechanism of PAAs has long been considered hydrolytic and internally catalyzed by the chain tert-amine groups located in the γ-position to the chain amide groups [1,2]. This hypothesis was apparently supported by the fact that the degradation occurred at pH > 7 where the PAA chain tert-amine groups were mostly unprotonated and, therefore, capable of exerting their catalytic activity. However, an alternative mechanism could have been the retro-aza-Michael reaction, since it is well-known that the Michael reaction is an equilibrium reaction much influenced by concentration. To ascertain this point, a small library of six PAAs and three glycine-based PAACs was expressly synthesized and their degradation on time at different pH values in dilute solution monitored by ^1^H-NMR. For the sake of simplicity, in this paper both linear PAAs and PAACs will be collectively referred to as PAAs. Evidence was collected that retro-aza-Michael reaction occurred, with qualifications. Besides pH, the degradation rate depended to some extent on the structure of both the parent amine- and bisacrylamide monomers. Overall, the results reported here shed light on the degradation mechanism of linear PAA in simple model environments, providing the basis for interpreting their degradation behavior in complex aqueous environments, such as biological fluids and moist soil, and establishing a rationale for interpreting the toxicity and ecotoxicity of their degradation products.

## 2. Materials and Methods

Materials. Solvents and reagents, unless otherwise specified, were analytical grade commercial products and used as received. *N,N’*-Methylenebisacrylamide (M, 99%), glycine (GLY, 98%), ethylamine (EA, aqueous solution at 70%), 2-methylpiperazine (MP, 95%), and lithium hydroxide monohydrate (LiOH^.^H_2_O, 98%) were purchased from Sigma-Aldrich (Milano, Italy) and used as received. 2,2-Bis(acrylamido)acetic acid (B) [34] and *N,N’*-bis(acryloyl)piperazine (BP) [35] were synthesized as previously described. Ultrafiltration was performed with regenerated cellulose membranes (molecular weight cut off 100 kDa and 5 kDa) in an Amicon^®^ Stirred Cell (Merck Italia, Milano, Italy) as PAA solutions in ultrapure water (18 MΩ·cm^−1^) produced with a Millipore Milli-Q^®^ apparatus (Darmstadt, Hesse, Germany). 

Characterizations. All polymers were characterized by ^1^H-NMR spectroscopy, using a Brüker Avance DPX-400 NMR spectrometer (Milano, Italy) operating at 400.13 MHz. Number of scans 32, relaxation delay, *d1*, 10.0 s, receiver gain automatically measured and set by the instrument. Analyses were conducted in D_2_O, adjusting the pH with D_2_O solutions of DCl or NaOD. 

All polymers were analyzed by attenuated total reflectance (ATR) Fourier transform infrared spectroscopy (FT-IR). FT-IR/ATR spectra were recorded at room temperature, in the 4000–380 cm^−1^ wavenumber range, with 32 scans and 4 cm^−1^ resolution using a Perkin-Elmer Frontier FT-IR/FIR spectrophotometer (Milano, Italy), equipped with a diamond crystal characterized by a penetration depth of 1.66 μm.

Size exclusion chromatography (SEC) traces were obtained with Toso-Haas TSK-gel G4000 PW and TSK-gel G3000 PW columns connected in series, using a Waters model 515 HPLC pump (Milano, Italy) equipped with a Knauer autosampler 3800 (Knauer, Bologna, Italy), a light scattering (670 nm, Malvern, Roma, Italy) and a refractive index detector (Model 2410, Waters, Milano, Italy). Mobile phase: 0.1 M Tris buffer pH 8.00 ± 0.05 and 0.2 M sodium chloride. Sample concentration: 20 mg mL^−1^; flow rate: 1 mL min^−1^; injection volume: 20 µL; loop size: 20 µL; column dimensions: 300 × 7.5 mm^2^. The instrument optical constants were determined using a narrowly distributed PEO 24 kDa standard. Before analysis, each sample was filtered through a 0.2 µm Whatman^TM^ syringe filter (Maidstone, UK). 

Synthesis of PAAs. All reactions were performed in a vessel equipped with a magnetic stirrer and nitrogen inlet/outlet. First, the amine monomer and, in the case of glycine, one mole lithium hydroxide monohydrate per mole amine were dissolved in water under inert atmosphere. Then, one mole bisacrylamide and, in the case of 2,2-bis(acrylamido)acetic acid, one further mole lithium hydroxide monohydrate was added and the system, after carefully purging with nitrogen, was warmed up to 50 °C under gentle stirring until complete solubilization. The reaction mixture was then maintained in the dark at 25 °C for 5 days. After this time, it was diluted to 50 mL with water, acidified to pH 3.5–4.0 with 37% HCl and then ultrafiltered in two steps through membranes with, respectively, molecular weight cut-off 100 kDa and 5 kDa using a 300 mL total volume of water for each step. The final product was recovered as an off-white powder by freeze-drying the solution passed through in the first ultrafiltration step but retained in the second ultrafiltration step. Yields 70–75%. The amounts of reagents and water used for each polymer preparation are reported in Table 1.

The FT-IR/ATR of the polymers are reported in Figures S1–S3 (Supplementary Materials) and the ^1^H-NMR spectra in Figure 1, Figure 2 and Figure 3 and Figures S4–S28.

Degradation studies by ^1^H-NMR at variable pH. All experiments were carried out at pH 4.0, 7.0 and 9.0 as 43.6 μM D_2_O solutions, calculated on the repeat units. The polymer amounts used are shown in Table 2. The pH of interest was then adjusted with 7% DCl or 10% NaOD in D_2_O. Each freshly prepared solution was introduced directly into a 5 mm Wilmad^®^ NMR tube (Merck KGaA, Darmstadt, Germany). The tube was then sealed, thermostated at 25 °C and analyzed by ^1^H-NMR at intervals. The amounts of the polymer samples used in the degradation experiments are reported in Table 2.

Degradation studies by ^1^H-NMR at 50 °C. The experiments were carried out at 50 °C and pH 9.0 with M-GLY using the polymer amount reported in Table 2 and following the same procedures as in the experiments at variable pH.

## 3. Results

### 3.1. Synthesis of PAAs 

**Scheme 1:**
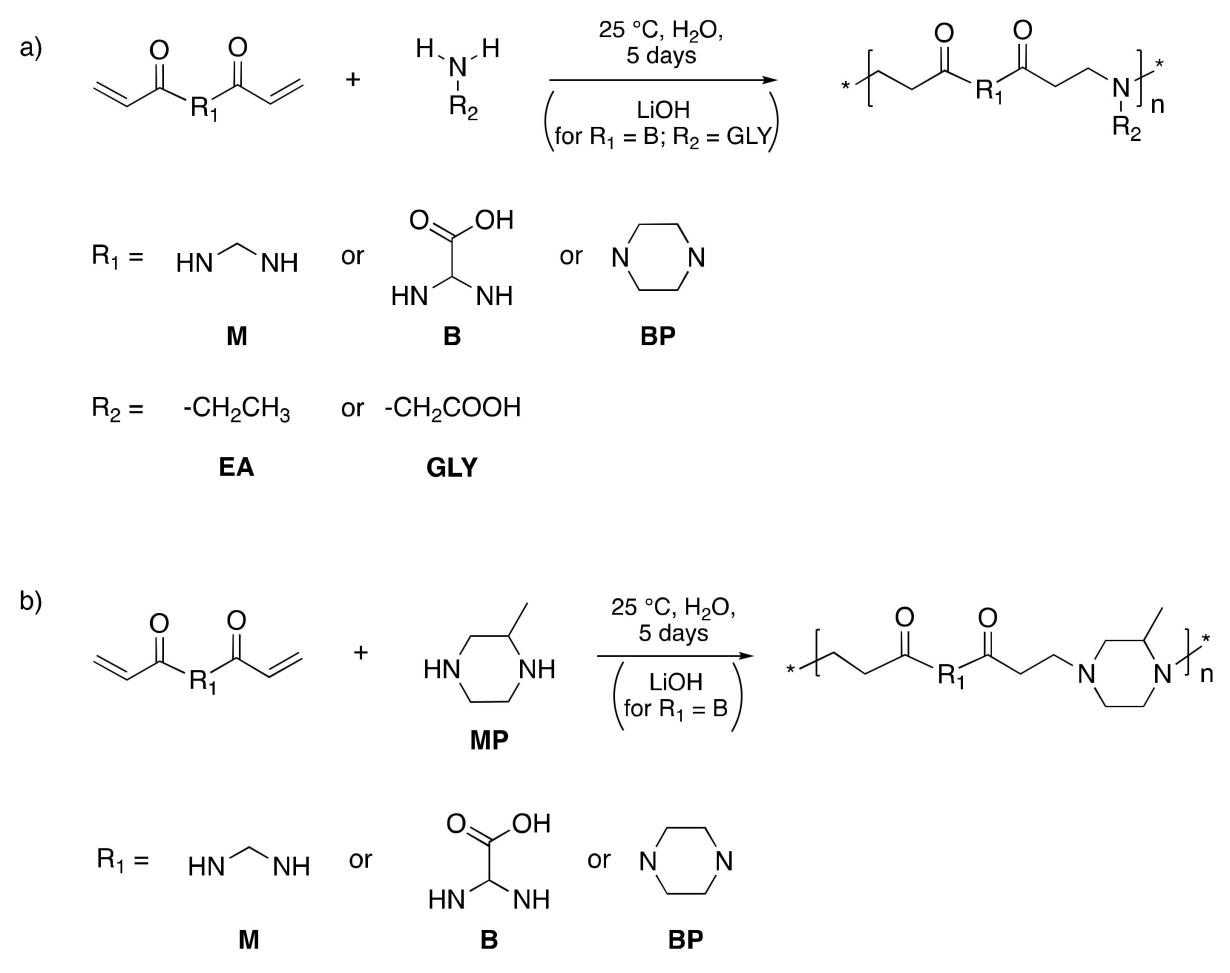
Synthesis of linear PAAs using a prim- (**a**) or bis-sec-amine (**b**) monomer.

A small library of nine linear PAAs was prepared by the combination of three bisacrylamides, namely *N,N’*-methylenebisacrylamide (M), 2,2-bis(acrylamido)acetic acid (B) and *N,N’*-bis(acryloyl)piperazine (BP) with three amines, that is, a prim-amine, namely ethylamine (EA), an α-amino acid, namely glycine (GLY) and a cyclic bis-sec-amine, namely 2-methylpiperazine (MP) (Table 1 and Table 3). The presence, among the monomers, of a carboxylated amine and a carboxylated bisacrylamide was in order to ascertain whether the presence of ionizable carboxyl groups influenced the degradability of the resulting polymers. The polymerization reaction was the aza-Michael polyaddition of amines (donors) with bisacrylamides (acceptors) carried out in water at pH ≥ 10 and 25 °C for 5 days at concentrations in the range 30–40 wt % (Scheme 1). No added catalysts were needed, since the amine monomers were sufficiently basic to self-catalyze the reaction. All polymers studied were completely soluble in water in the pH range of interest for the present work.

The structures of the repeat units of all polymers (Table 3) were confirmed by FT-IR (Figures S1–S3) and ^1^H-NMR (D_2_O, pH 4.0, Figure 1 and Figures S4–S11) spectroscopies.

The molecular weights of the PAAs studied, determined by SEC with LALLS detector, are reported in Table 4. It can be observed that they were not homogeneous since each pair of monomers had different reaction kinetics. However, it was preferred to prepare and isolate all polymers following the same procedures instead of fractionating them extensively to bring them to similar molecular weights. 

### 3.2. Degradation in Water of Linear PAAs by ^1^H-NMR

**Scheme 2:**
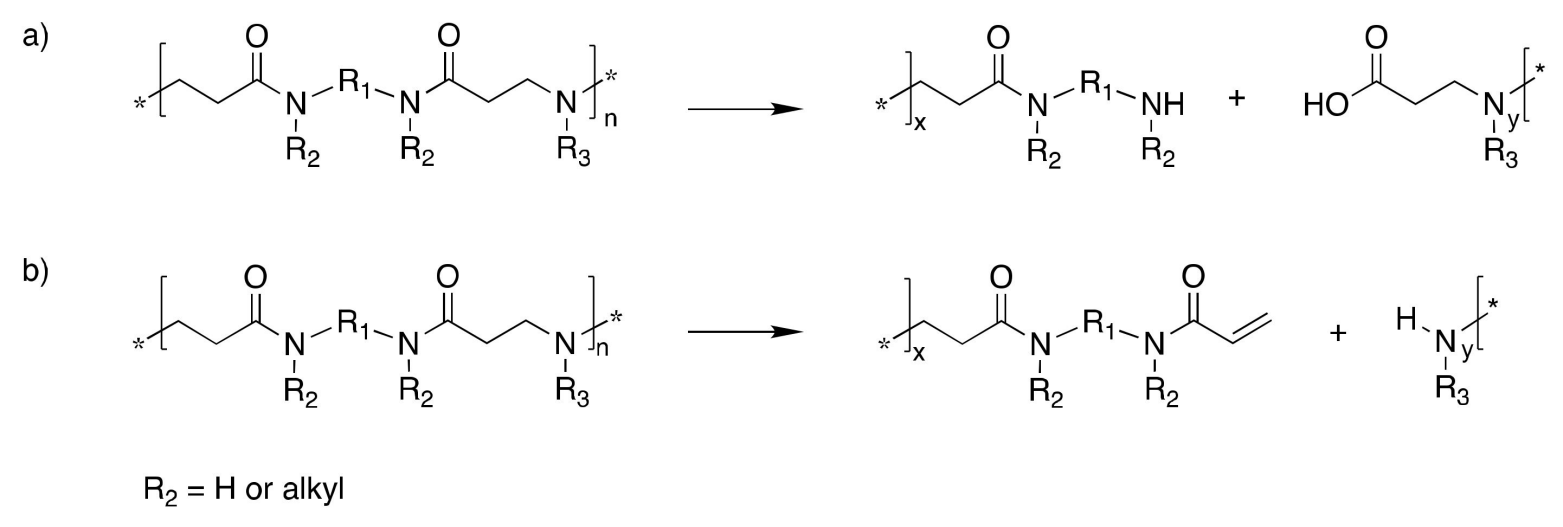
PAA degradation by: (**a**) Amide bond hydrolysis; (**b**) retro-aza-Michael.

The degradation mechanism of PAAs in water was studied by ^1^H-NMR with the aim of identifying the chain terminals produced following degradation. Two different degradation mechanisms could be hypothesized: The hydrolytic cleavage of the amide bonds (Scheme 2a) and the retro-aza-Michael de-polymerization (Scheme 2b). The former gives rise, for each cleaved bond, to a carboxylate- and either a prim- or a sec-amine terminal, depending on the nature of the acrylamide moiety, whereas the latter produces in all cases, for each dissociation step, a sec-amine- and an acrylamide terminal.

The pH-dependence of degradation was first investigated by carrying out experiments at pH 4.0, 7.0 and 9.0, keeping in all experiments the same repeat unit concentration (43.6 μM) and temperature (25 °C) (Table 2). The temperature dependence was studied using M-GLY as model at pH 9.0 and 50 °C. Degradation experiments were always conducted in sealed NMR tubes maintained at 25 °C (in the case of M-GLY also at 50 °C) and analyzed at intervals. The results (Section 3.2.1, Figure 4 and Figure 5) were reported as the increase over time of the percentage of the repeat units that underwent degradation, in turn determined from the ratio of the newly formed terminal groups over the originally present units (see captions to Figure 2 and Figure 3 and Figures S12–S27). 

#### 3.2.1. Degradation Experiments at Different pH’s 

**Figure 2:**
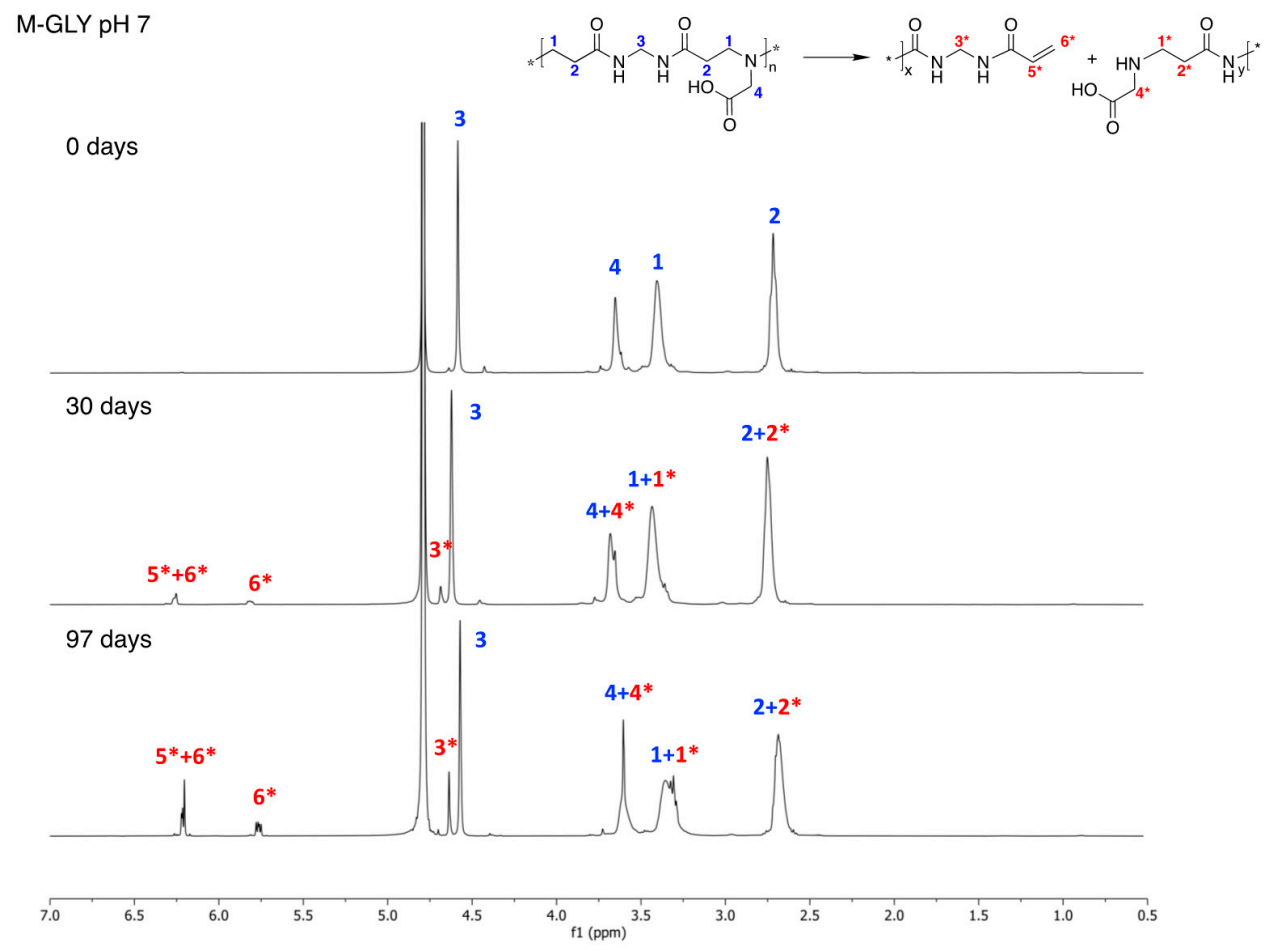
^1^H-NMR spectra of M-GLY obtained in D_2_O at pH 7.0 at different times using a 400 MHz Avance Brüker spectrometer. Concentration of the repeat unit 43.6 μM. Degradation percent calculated both as [H_3*_/(H_3_ + H_3*_)] × 100 and as {[(H_5*_ + H_6*_)/3]/[(H_2_ + 2H_2*_)/4]} × 100, where H_n_ stands for the integral of the resonance peak of the hydrogen indicated in the subscript.

The ^1^H-NMR spectra at pH 4.0 and 25 °C were collected for all polymers over a period of 99 days (Figure 2 for M-GLY and Figures S4–S11 for all the other polymers). Some structural variations were observed for BP-EA (Figure S9), whereas the spectra of all the other polymers did not change over the entire duration of the experiment, proving that in these conditions they were stable up to 99 days and probably more. As for BP-EA, three barely discernible peaks initially present in its ^1^H-NMR spectrum at 5.77, 6.21 and 6.75 ppm (6* and 7* in Figure S9), attributed to traces of residual double bonds, slightly increased throughout all the duration of the experiment and, correspondingly, three novel peaks at 1.31, 2.88 and 3.15 ppm (5*, 2* and 4* in Figure S9), attributed to terminals consequent to the retro-aza-Michael reaction, respectively C**H**_3_–CH_2_–, C**H**–CON– and C**H**_2_–NH–, appeared. This indicated that for BP-EA the retro-Michael reaction took place even at pH 4.0, albeit to a very slow rate (3% after 99 days). 

The ^1^H-NMR spectra at pH 7.0 and 25 °C were recorded for all polymers over a period of 97 days. For all samples, the occurrence of the retro-aza-Michael reaction was demonstrated by the appearance and the progressive increase of the proton resonance peaks ascribed to two types of terminals, i.e., the acrylamide terminals, revealed by the C**H**_2_=C**H** hydrogens (5* and 6* in Figure 2) and the sec-amine terminals, revealed for instance in M-GLY by the newly formed HN–C**H**_2_–COO^−^ hydrogens (4* in Figure 2) (see captions to Figures S12–S19 for all the other polymers). No new peaks specifically attributable to hydrolytic degradation products were observed. 

The progress of the retro-aza-Michael reaction was followed by considering the polymer repeat units as the reacting species. It was quantitatively determined by calculating the ratio, at different times, of the integral due to a single hydrogen belonging to a newly formed terminal over the integral due to a single hydrogen in the originally present repeat unit (see captions of Figure 2 and Figures S12–S19). The kinetic curves of the retro-aza-Michael degradation at pH 7.0 of all polymers are shown in Figure 4. 

Apart from BP-GLY and BP-EA, degradation experiments were consistent with first order kinetics, studied by considering the polymer repeat units as the reacting species, confirming that the by far prevailing degradation mechanism was the retro-aza-Michael reaction (see Discussion). The related kinetic rate constants are reported in Table 5. In some instances (M-EA and B-EA), the experimental percent degradation at 97 days was somewhat lower than the value determined by extrapolating the fitting curve. As for BP-GLY, the percent degradation reached nearly 40% after 15 days and then apparently decreased monotonically till the end of the experiment, suggesting a competing reaction (see Discussion).

From the data of pH 7.0 in Table 5, it appears that the main effect on the rate of degradation was exerted by the structure of the amine portion of the repeat unit. The structure of the amide portion also influenced the reaction rate, but to a lesser extent. In fact, irrespective of the bisacrylamide structure, the kinetic rate constants of the retro-aza-Michael reaction were in the order: EA-based polymers >> GLY-based polymers > MP-based polymers. The experimentally determined degradation percentages at 97 days followed the same order. As for the influence of the bisacrylamide moiety, the degradation rates were in the order: BP-based polymers >> M-based polymers > B-based polymers. 

**Figure 3:**
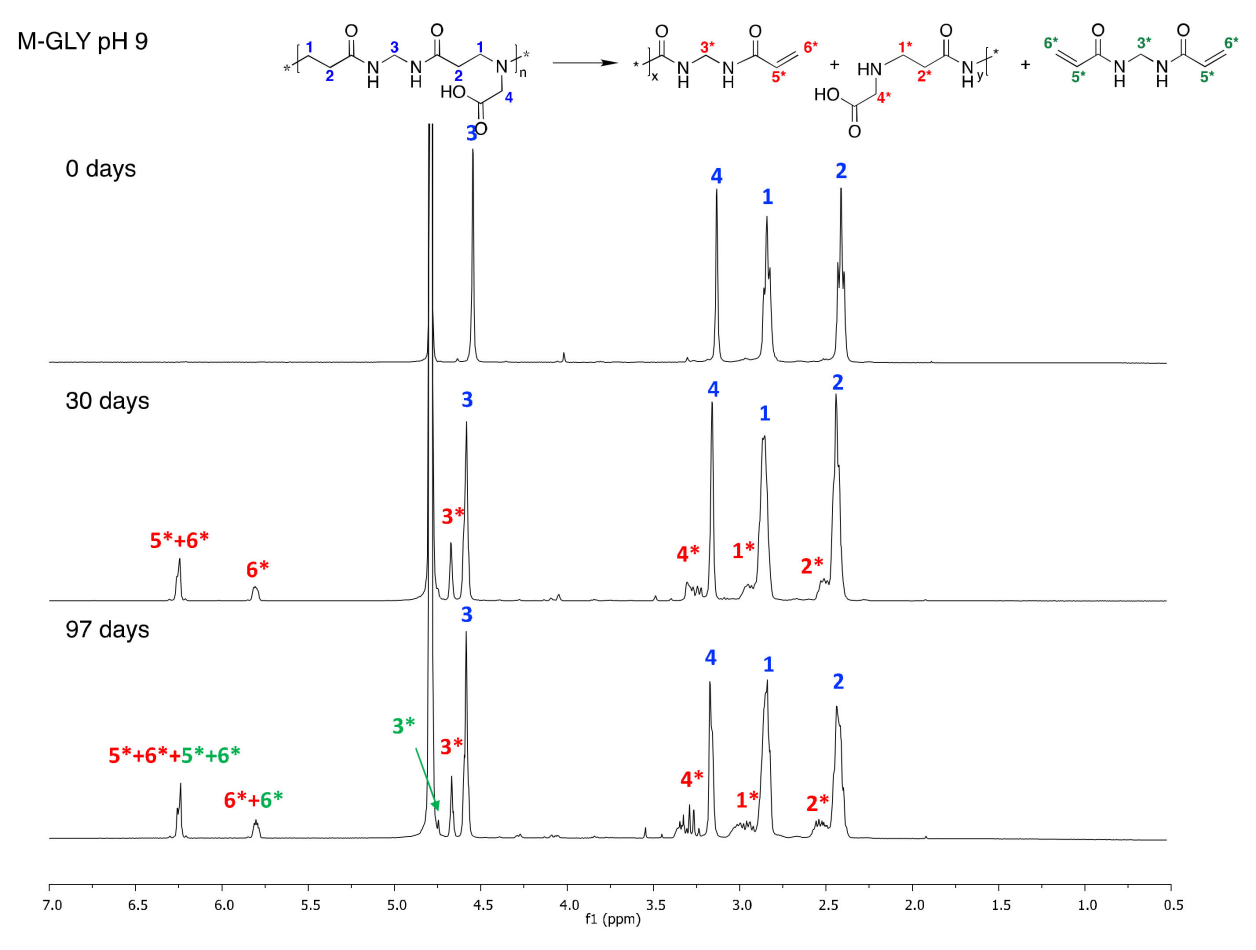
^1^H-NMR spectra of M-GLY obtained in D_2_O at pH 9.0 and different times using a 400 MHz Avance Brüker spectrometer. Concentration of the repeat unit 43.6 μM. Degradation percent calculated both as [H_3*_/(H_3_ + H_3*_)] × 100 and as {[(H_5*_ + H_6*_)/3]/[(H_2_ + 2H_2*_)/4]} × 100 and as [H_1*_/(H_1_+2H_1*_)] × 100, where H_n_ stands for the integral of the resonance peak of the hydrogen indicated in the subscript. Multiple 1*, 2* and 4* peaks are possibly related to different ionization states of the sec-amine terminal groups.

The ^1^H-NMR spectra at pH 9.0 and 25 °C were recorded for all polymers at intervals over a period of 97 days. Moreover, in this case, significant retro-aza-Michael reaction was revealed by the appearance of peaks ascribable to the acrylamide- and the sec-amine terminals, as shown in Figure 3 for M-GLY and in Figures S20–S27 for all the other polymers. The kinetic curves of the retro-aza-Michael degradation at pH 9.0 of all polymers, obtained as previously described for experiments at pH 7.0, are shown in Figure 5. The kinetic curves increased monotonically throughout the observation time only for B-GLY, B-MP and BP-MP. For all the other polymers, the kinetic curves reached a plateau at different intermediate times (15 days for M-EA and M-MP, 40 days for M-GLY). As for BP-GLY, whose data were obtained only considering the vinyl terminals, the degradation degree reached nearly 40% after 15 days and then, as also observed at pH 7.0, decreased monotonically till the end of the experiment. For BP-EA the curves obtained considering the vinyl- or the sec-amine terminals showed different trends (see Discussion). As for the influence of the amine moieties, overall the maximum degradation degree was in the order: GLY-based polymers ≥ EA-based polymers >> MP-based polymers. In fact, the degradation degree at 97 days ranged from < 10% for MP-polymers, to 10–38% for EA-polymers, to 20–40% for GLY-polymers. The latter observation, together with the overall faster degradation of the B-based polymers compared with the M-based ones, suggested a role of the carboxylate group in facilitating the retro-aza-Michael reaction at pH 9.0. 

#### 3.2.2. Degradation of M-GLY at 50 °C 

It is well known that the activation energy of the reverse aza-Michael reaction is higher than that of the direct reaction [36]. Therefore, it could be expected that higher temperatures would speed up the degradation reaction. This was investigated with a spot-wise experiment carried out with M-GLY at pH 9.0 and 50 °C. It was observed that the NMR spectra of the degrading M-GLY at pH 9.0 and 50 °C (Figure S28) did not significantly differ from those recorded at the same pH and 25 °C (Figure 3), confirming that the nature of the degradation products was similar in both cases. The results of the experiment at 50 °C compared with those obtained at 25 °C (Figure 6) suggested that at 50 °C the retro-aza-Michael reaction started considerably faster, but at longer reaction times apparently receded. This is in line with the occurrence of a hydrolytic degradation that was, of course, favored by the higher temperature. 

## 4. Discussion

The present work was aimed at demonstrating experimentally that the degradation of PAAs in dilute solution is mainly due to retro-aza-Michael and, in the meantime, at estimating the influence on the degradation rate both of the polymer structure and of external factors such as pH and temperature.

**Scheme 3:**
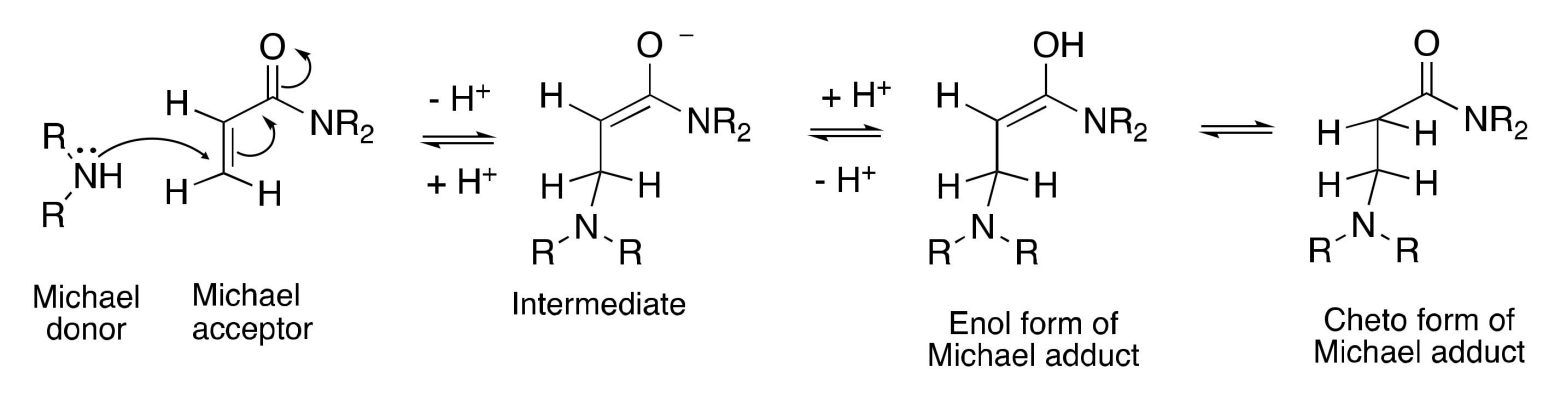
Mechanism of the aza-Michael addition reaction of a sec-amine to an acrylamide.

The Michael reaction is the 1,4-addition of a nucleophile to the β-carbon atom of an α,β-unsaturated compound bearing an electron-withdrawing group (EWG). Protic solvents, such as water, promote rapid proton transfer and stabilize charged intermediates. When the Michael reaction involves nitrogen-donors, including prim- or sec-amines, it is referred to as aza-Michael [37]. When the aza-Michael reaction involves an acrylamide, the recognized mechanism is that reported in Scheme 3.

Amines can behave both as nucleophiles and bases and, therefore, the aza-Michael reaction can also occur in the absence of added basic catalysts. Among the factors that influence the reactivity of aliphatic amines, nucleophilicity and steric hindrance are of fundamental importance. These elements may conflict: Dibutylamine, notwithstanding its higher nucleophilicity, reacts slower than aliphatic prim-amines [38]. Prim-amines can undergo double aza-Michael addition [39]. However, the second addition step is usually slower, due to steric hindrance. Cyclic sec-amines are stronger nucleophiles than prim-amines due to the inductive effect of the *N*-substituted alkyl groups and are normally less hindered than linear sec-amines of similar composition; therefore, they usually react faster as Michael donors [40]. However, when alkyl substituents are present in vicinal positions to the NH group, such as for instance in 2-methylpiperazine and 2,4-dimethylpiperazine, the reaction is slower [41].

The aza-Michael reaction, widely exploited for the synthesis of linear polymers [3,42], hyperbranched polymers [43,44], dendrimers [45,46,47] and cross-linked hydrogels [48,49], is thermodynamically controlled and reversible. The aza-Michael/retro-aza-Michael equilibrium has indeed been exploited for synthetic purposes [50,51,52]. It is noteworthy that the Michael addition is bimolecular, whereas the reverse reaction is monomolecular. Therefore, they follow different kinetics and respond differently to environmental stimuli. For instance, the aza-Michael/retro-aza-Michael equilibrium position is shifted in favor of aza-Michael by concentration and in the opposite direction by dilution. This fact considered, and based on previous experience, in this work the overall monomer concentration used for preparing the PAAs of interest was ~3 M in water. This concentration allowed obtaining the molecular weights reported in Table 4. Broadly speaking, the polymerization degree of PAAs achievable in given conditions indicate their aza-Michael/retro-aza-Michael equilibrium positions in these conditions. The particularly high molecular weights of PAAs based on MP, a cyclic bis-sec-amine, were probably related to the fact that this amine was more reactive in the direct reaction. 

Based on this premise, one could reasonably expect that aqueous solutions of PAAs more diluted than their polymerizing mixtures could induce degradation by shifting their equilibrium positions towards the retro-aza-Michael reaction. 

The aza-Michael/retro-aza-Michael equilibrium is pH-dependent because involves proton exchange. In this work, degradation experiments were performed at pH values below that normally adopted in PAA synthesis (pH 10–11), where the amine groups of both monomers and repeat units are deprotonated. The pH values considered in this work were 4.0, 7.0 and 9.0. At pH 4.0, where all amine groups were protonated (Table 6) and, moreover, the tautomeric enolate/enol equilibrium (Scheme 3) was completely shifted towards the enol form, no degradation was observed for nearly all systems, apart from BP-EA, which showed little degradation (3%) after 99 days (Figure S9). By contrast, at pH 7.0 and 9.0, significant retro-aza-Michael degradation occurred for all systems, even if slightly so for M-MP at pH 9.0 (Figure S21). At these pH’s, the tert-amine groups in the polymer chains were only partially protonated (α = 0.56 at pH 7.0, Table 6) or little protonated (α = 0.01 at pH 9.0, Table 6), whereas at the same pH’s the starting amine monomers were fully or largely protonated (see Table 6). By analogy, the sec-amine terminals generated by retro-aza-Michael were most probably more basic than the tert-amine groups in the polymer chain. As a consequence, at pH 7.0 and 9.0 the retro-aza-Michael products were more protonated and acted as leaving groups since in the protonated state withdrew from the aza-Michael/retro-aza-Michael equilibrium shifting it towards dissociation. 

**Figure 4:**
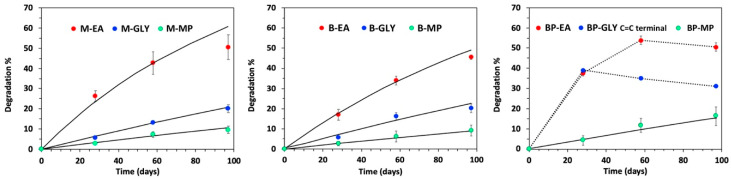
Degradation kinetics of PAAs at pH 7.0 and 25 °C. Concentration of the repeat unit 43.6 μM. Each panel shows the degradation curves relative to polymers obtained from the same bisacrylamide. The solid circles, with the related error bars, represent the experimental data determined as the averages of the values obtained considering different terminal- and internal repeat unit reference peaks (see also the captions to Figure 2 and Figures S12–S19). The continuous lines are the fitting curves calculated by using the first order kinetic constants of Table 5. The dotted lines (BP-EA and BP-GLY) refer to trendlines not following first order kinetics.

**Figure 5:**
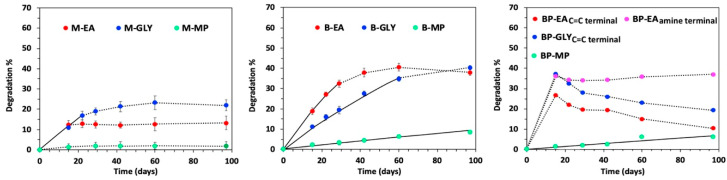
Degradation kinetics of linear PAAs at pH 9.0 and 25 °C. Concentration of the repeat unit 43.6 μM. Each panel groups the degradation curves relative to polymers obtained from the same bisacrylamide. The solid circles, with the related error bars, represent the experimental data determined as the averages of the values obtained considering different terminal- and internal repeat unit reference peaks (see also the captions to Figure 3 and Figures S20–S27). The continuous lines are the fitting curves calculated by using the first order kinetic constants of Table 5. The dotted lines refer to trendlines not following first order kinetics.

**Figure 6:**
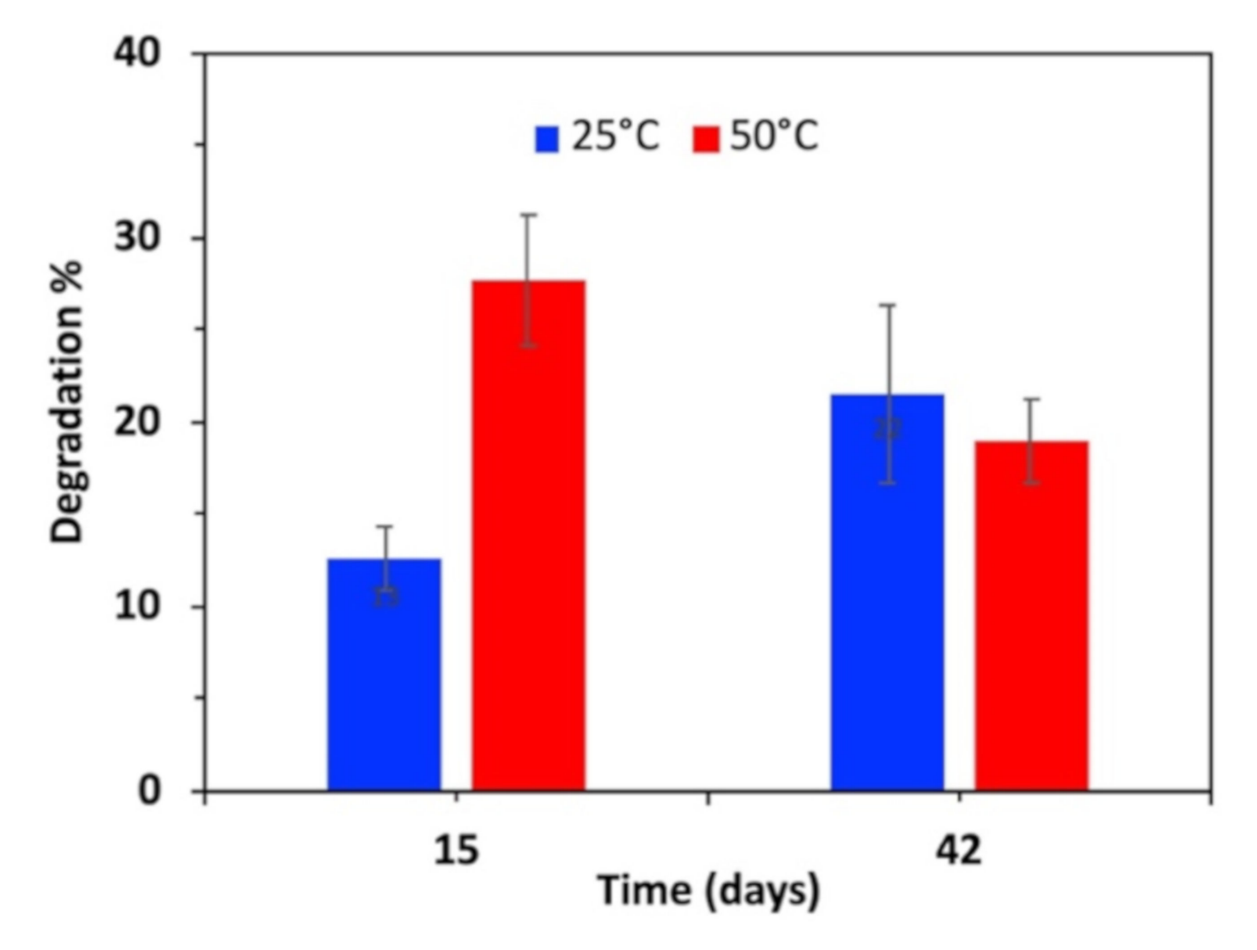
Temperature dependence of M-GLY degradation at pH 9.0.

The different amine basicity provides a possible explanation of why both at pH 7.0 and 9.0 the MP-polymers were remarkably less degradable than GLY- and EA-polymers (Figure 4 and Figure 5). In fact, the repeat units of MP-polymers were scarcely protonated compared with those of GLY- and EA-polymers. This likely disfavored the final dissociation step of the tert-amine in the retro-aza-Michael reaction. As for the effect of the amide moieties, this was less significant than that of the amine moieties, particularly so at pH 7.0. At pH 9.0, the M-based polymers were the most stable (Figure 5). 

As previously observed, the molecular weights of the polymer samples considered were not homogeneous, ranging from 34,000 (B-MP) to 5500 (B-GLY). No apparent connection between the sample molecular weights and their degradation rates was observed. The degradation rate was mostly dependent on the structure of the repeat unit. This made it unlikely the hypothesis that degradation followed an unzipping mechanism. Unzipping would have produced significant amounts of free monomers from the early stages of the reaction. On the opposite, traces of bisacrylamide monomer were only observed in the ^1^H-NMR spectra of M-EA at pH 7.0 and 9.0 (Figures S12 and S20) and M-GLY at pH 9.0 (Figure 3) at advanced stages of reaction. 

**Figure 7:**
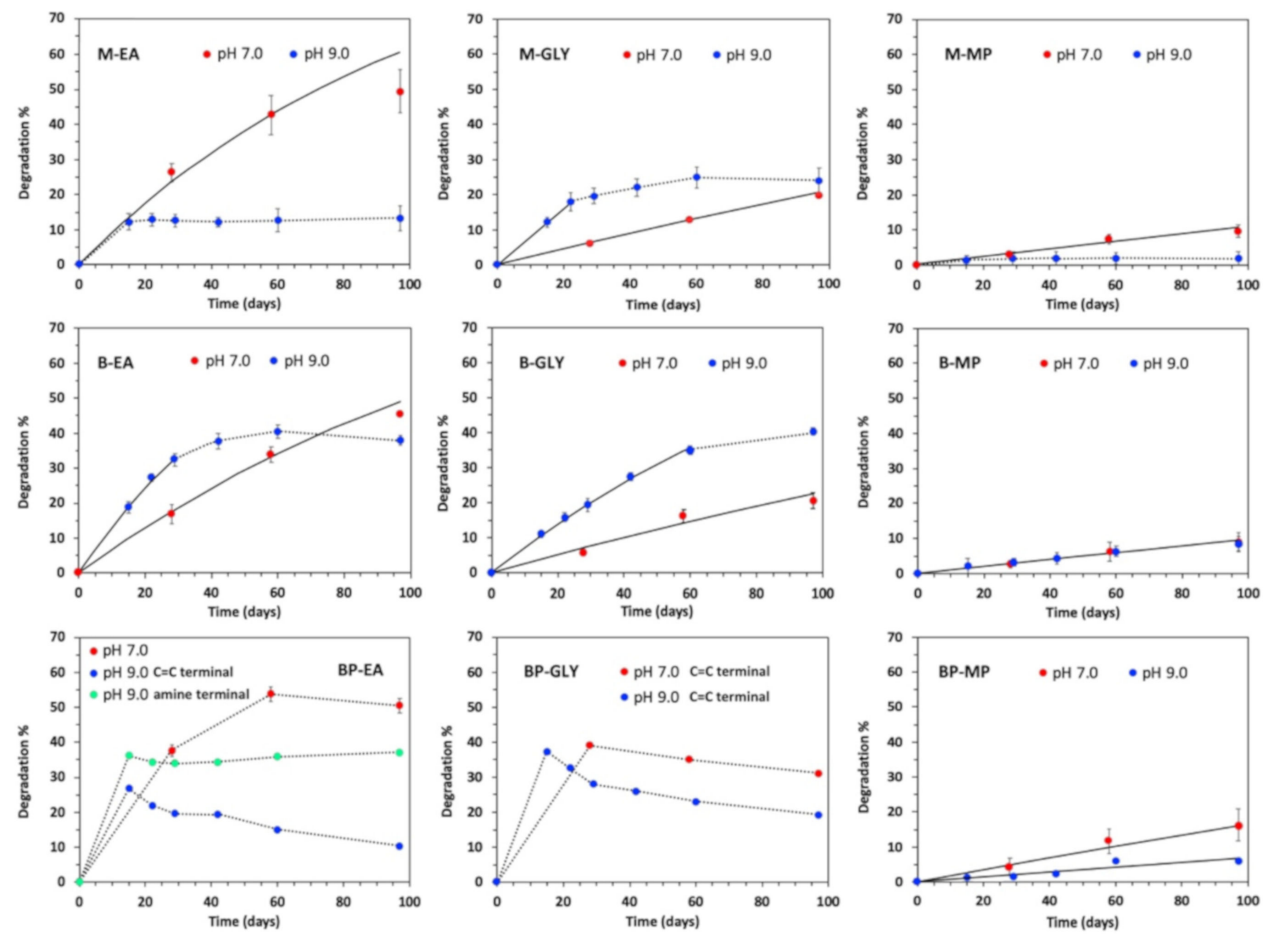
Comparison of the degradation behavior of linear PAAs at pH 7.0 and 9.0.

It may be observed that at pH 7.0 the degradation normally started more slowly than at pH 9.0 (see comparisons in Figure 7) and in most cases increased progressively following first order kinetics. In the case of M-EA and B-EA, the experimental percent degradation at 97 days was lower than the value determined by extrapolating the fitting curve, possibly because the system approached the aza-Michael/retro-aza-Michael equilibrium under the degradation conditions, particularly at the experiment dilution. As for BP-EA, the degradation curve shown in Figure 4, obtained considering only the vinyl terminals, was not consistent with first order kinetics and, after reaching the maximum of 55% after 58 days, slightly decreased. It may be observed that in the ^1^H-NMR spectrum both the shape and the ratio of integral values of the hydrogen vinyl bonds (6* and 7* Figure S17) changed. In particular, two small peaks appeared at 5.77 and 6.23 ppm just superimposed to those of 7*, and the 6* hydrogen integral was apparently lower than those of 7* hydrogens. In contrast, the intensities of 1*, 2* and 4* peaks, ascribed to the sec-amine terminals, clearly increased until the 97th day, even if their quantification was practically impossible because these peaks partially overlapped to adjacent signals. As for BP-GLY, the kinetic curve obtained by monitoring the integral of the acrylamide terminals reached nearly 40% degradation after 15 days and then decreased monotonically till the end of the experiment. Moreover, in this case, the two small peaks at 5.77 and 6.23 ppm, observed in the degraded BP-EA spectra, were detected. These peaks were more visible in the BP-EA and BP-GLY spectra recorded during the degradation experiments at pH 9.0 and will be discussed in the relevant paragraph (see below). For both polymers, the progressive reduction of the acrylamide double bonds suggested the occurrence of a side reaction that produced groups capable of saturating a portion of these double bonds, thus reducing the apparent extent of the retro-aza-Michael reaction. The most probable hypothesis was the hydrolysis of some amide groups, which produced novel piperazine terminals that possibly underwent further aza-Michael reaction with the acrylamide terminals, as revealed by the appearance of two small peaks in the 2.50–2.70 ppm range (Figures S17 and S18).

As previously stated, in most cases at pH 9.0 the degradation rate started more rapidly than at pH 7.0, then slowed down reaching a plateau within 10 to 60 days (Figure 7), possibly because the equilibrium position was reached. This was not observed for B-MP and BP-MP, which degraded very slowly and did not apparently reach any plateau. As for BP-EA and BP-GLY, the integrals of the acrylamide hydrogens (6*, 7* and 5*, 6*, respectively), after reaching a maximum at 15 days, progressively decreased till the end of the experiment (Figures S25 and S26), whereas two equally intense small broad singlets at 5.80 and 6.20 ppm correspondingly increased and at 97 days prevailed. Analogously to what observed at pH 7.0, the disappearance of the acrylamide hydrogen signals was ascribed to the occurrence of the amide bond hydrolysis followed by a further aza-Michael addition by the newly formed piperazine groups. The fact that this phenomenon was more pronounced at pH 9.0 than at pH 7.0 supported this hypothesis because the amide hydrolysis is favored by higher pH’s. Furthermore, the two singlets at 5.80 and 6.20 ppm recall the pattern of geminal hydrogens of a substituted double bond and were tentatively attributed to radical reactions between the acrylamide terminals formed in each retro-aza-Michael step. Radical dimerizations are, for instance, the basis of spontaneous thermal and photochemical self-polymerization of vinyl monomers [57,58,59,60]. It may be observed that the degrading samples were purposely maintained for months in natural conditions of temperature and light.

## 5. Conclusions

This work shed light on the degradation mechanism of linear PAAs in aqueous solution, laying the foundations for interpreting their behavior in more complex aqueous degradation environments, as for instance the biological environment, wet soil, just to mention a few. The main finding was that all tested PAAs essentially degraded in dilute aqueous solution by undergoing retro-aza-Michael reaction. Following a first order kinetic, this mechanism is obviously triggered by dilution. For all of them, the degradation became evident in the pH interval 7.0–9.0, whereas it was almost negligible at pH 4.0, a decidedly acidic pH, where the polymer amine groups were completely protonated and the enolate/enol equilibrium was unfavorably affected. An unzipping depolymerization mechanism was deemed unlikely, since no significant amounts of free monomers were observed in the early stages of degradation, whereas traces of monomers were revealed in some instances by ^1^H-NMR only at high conversions. 

No spectral evidence of extensive hydrolytic degradation was found, but in several cases the kinetic curves provided clear hints that some hydrolytic cleavage of the amide bonds occurred along with the retro-aza-Michael reaction. This was particularly evident in the M-GLY experiment carried out at 50 °C. Interestingly, the comparison of the NMR spectra of M-GLY obtained at this temperature and pH 9.0 with those recorded at the same pH and at 25 °C suggested that the nature of the degradation products was similar both cases.

At pH 7.0, first order kinetic rate degradation constants could be calculated for most polymers, except for BP-EA and BP-GLY, which showed uneven degradation trends likely due to concurrent degradation mechanisms. At pH 9.0 the degradation normally proceeded faster than at pH 7.0 in the early stages of the experiment; therefore, it slowed down sharply once a certain percentage of degradation was reached. In most cases, this allowed to obtain well-defined kinetic rate constants only for the early segments of the degradation curve. The results obtained pointed to the conclusion that the degradation rates of PAAs roughly followed the order of the basicity of the amine monomers and, consequently, of the tert-amine groups in their repeat units. Those deriving from more strongly basic amines degraded faster most likely due to the higher degree of protonation of their repeat units that favored the last step of the retro-aza-Michael reaction. 

## Figures and Tables

**Figure 1:**
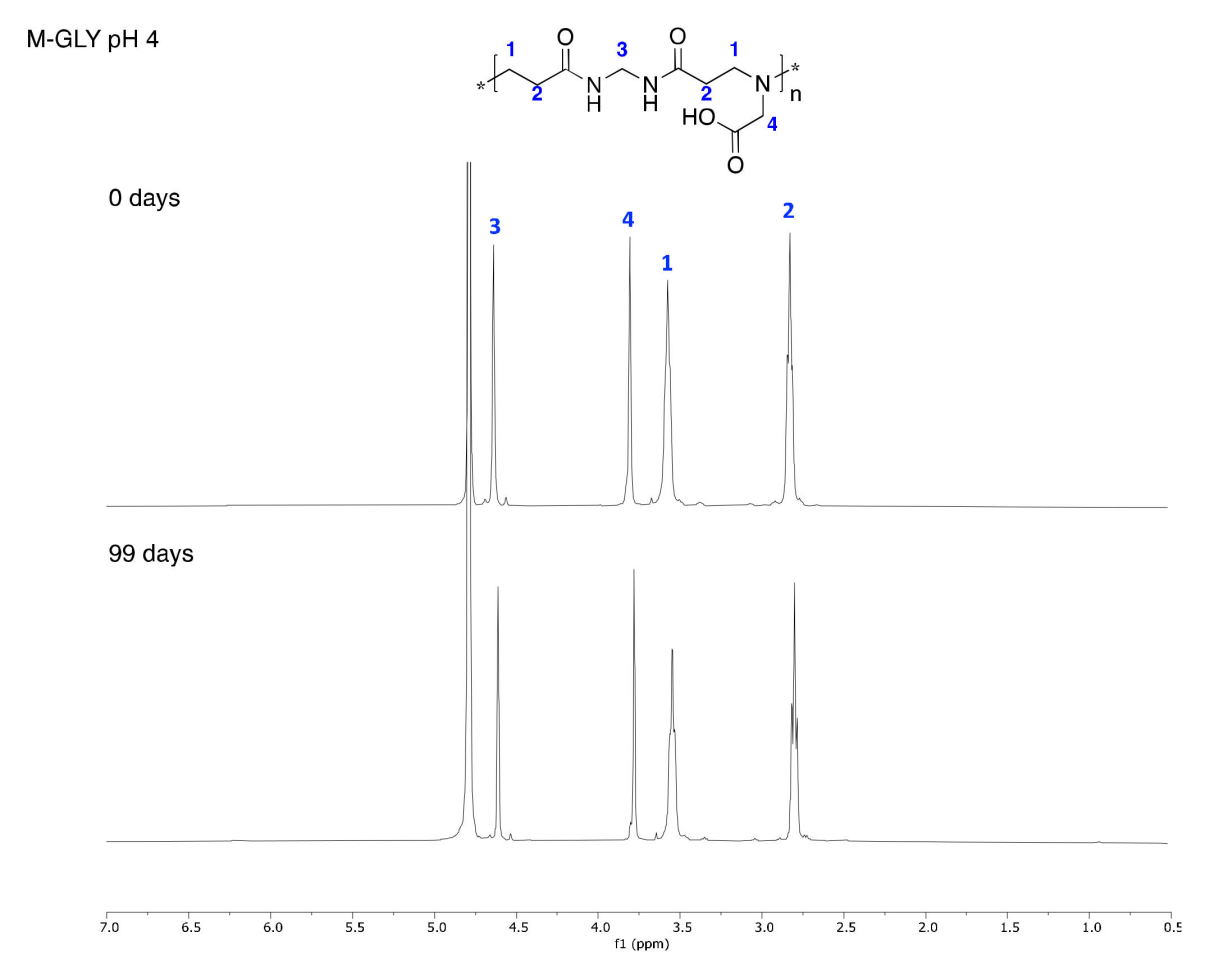
^1^H-NMR spectra of M-GLY obtained in D_2_O at pH 4.0 at different times using a 400 MHz Avance Brüker spectrometer. Concentration of the repeat unit 43.6 μM.

**Table 1:** Amounts of reagents used in polyamidoamine (PAA) syntheses.

Sample	Bisacrylamide Monomer		Amine Monomer		LiOH·H_2_O		H_2_O
	(g)	(mmol)	(g)	(mmol)	(g)	(mmol)	(mL)
M-EA	1.54	10	0.64	10	-		4.7
M-GLY	1.54	10	0.76	10	0.43	10	4.1
M-MP	1.54	10	1.05	10	-		3.9
B-EA	2.09	10	0.64	10	0.43	10	4.7
B-GLY	2.09	10	0.76	10	0.86	20	5.6
B-MP	2.09	10	1.05	10	0.43	10	5.4
BP-EA	2.00	10	0.64	10	-		4.0
BP-GLY	2.00	10	0.76	10	0.43	10	4.9
BP-MP	2..00	10	1.05	10	-		4.6

**Table 2:** Polymer amounts used in the ^1^H-NMR degradation studies at pH 4.0, 7.0 and 9.0 ^a^^,b^.

Sample	Sample Weight (mg)	Moles of Repeat Unit (μmol)
M-EA	8.7	43.6
M-GLY	10.0	43.6
M-MP	11.1	43.6
B-EA	10.6	43.6
B-GLY	11.9	43.6
B-MP	13.0	43.6
BP-EA	10.4	43.6
BP-GLY	11.7	43.6
BP-MP	12.8	43.6

^a^ Dissolved in 1 mL D_2_O; ^b^ pH adjusted with DCl or NaOD in D_2_O.

**Table 3:** Repeat units of the linear PAAs studied ^a^.

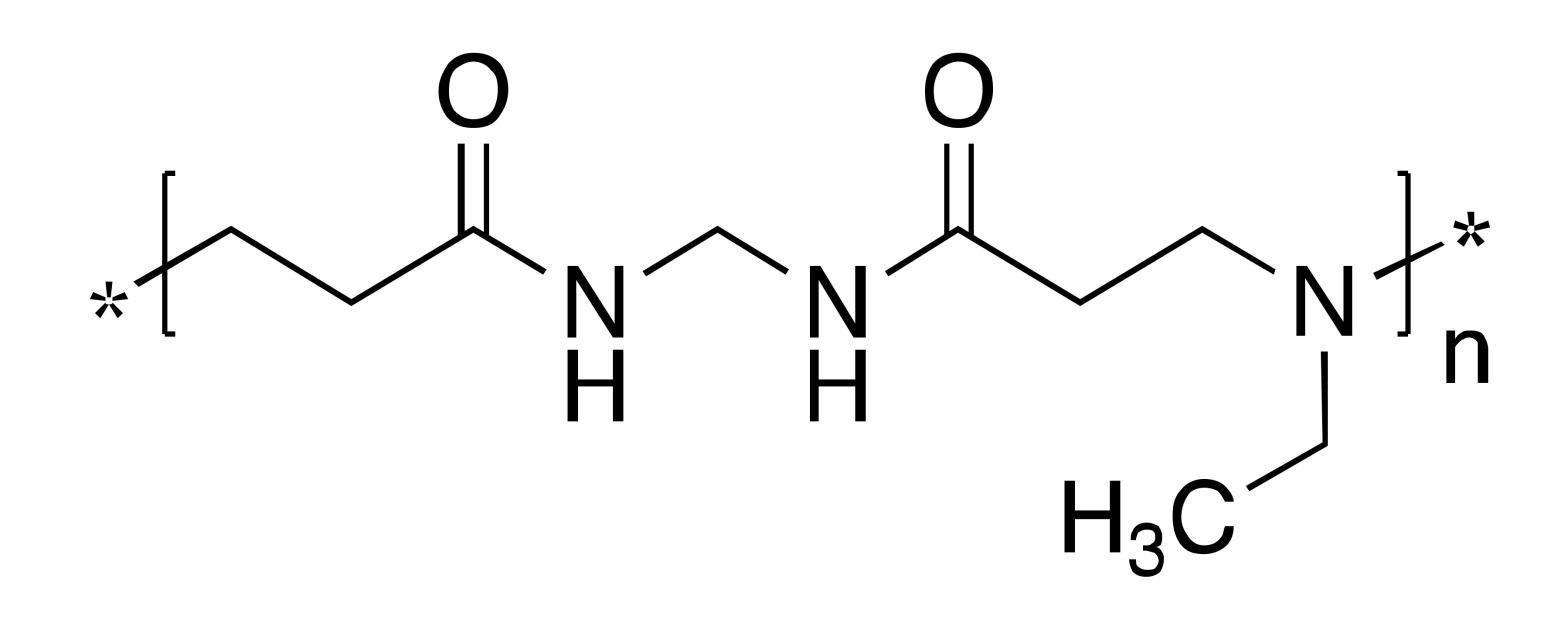	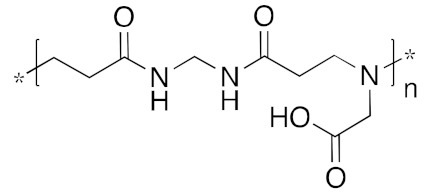	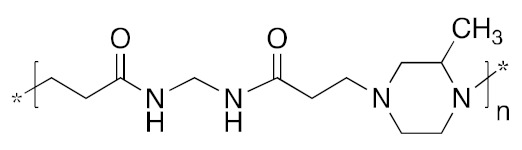
**M-EA**	**M-GLY**	**M-MP**
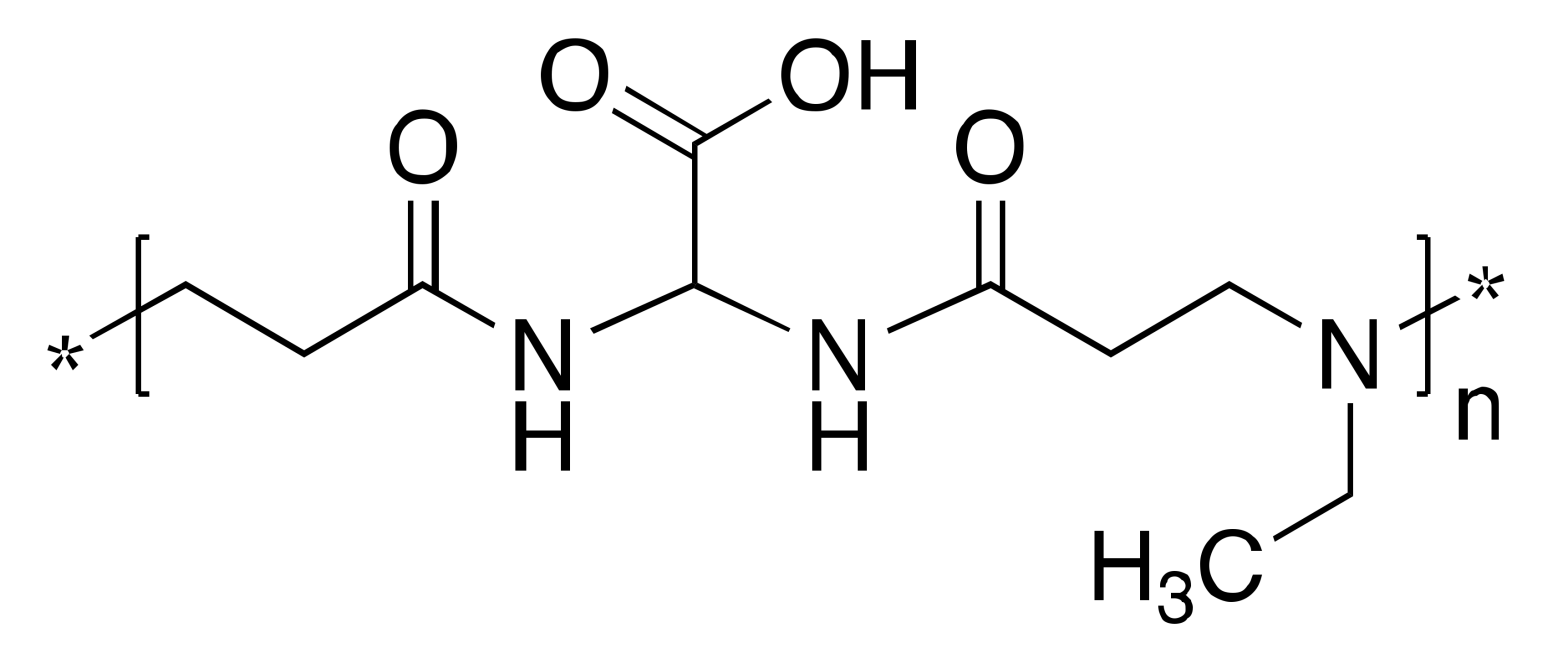	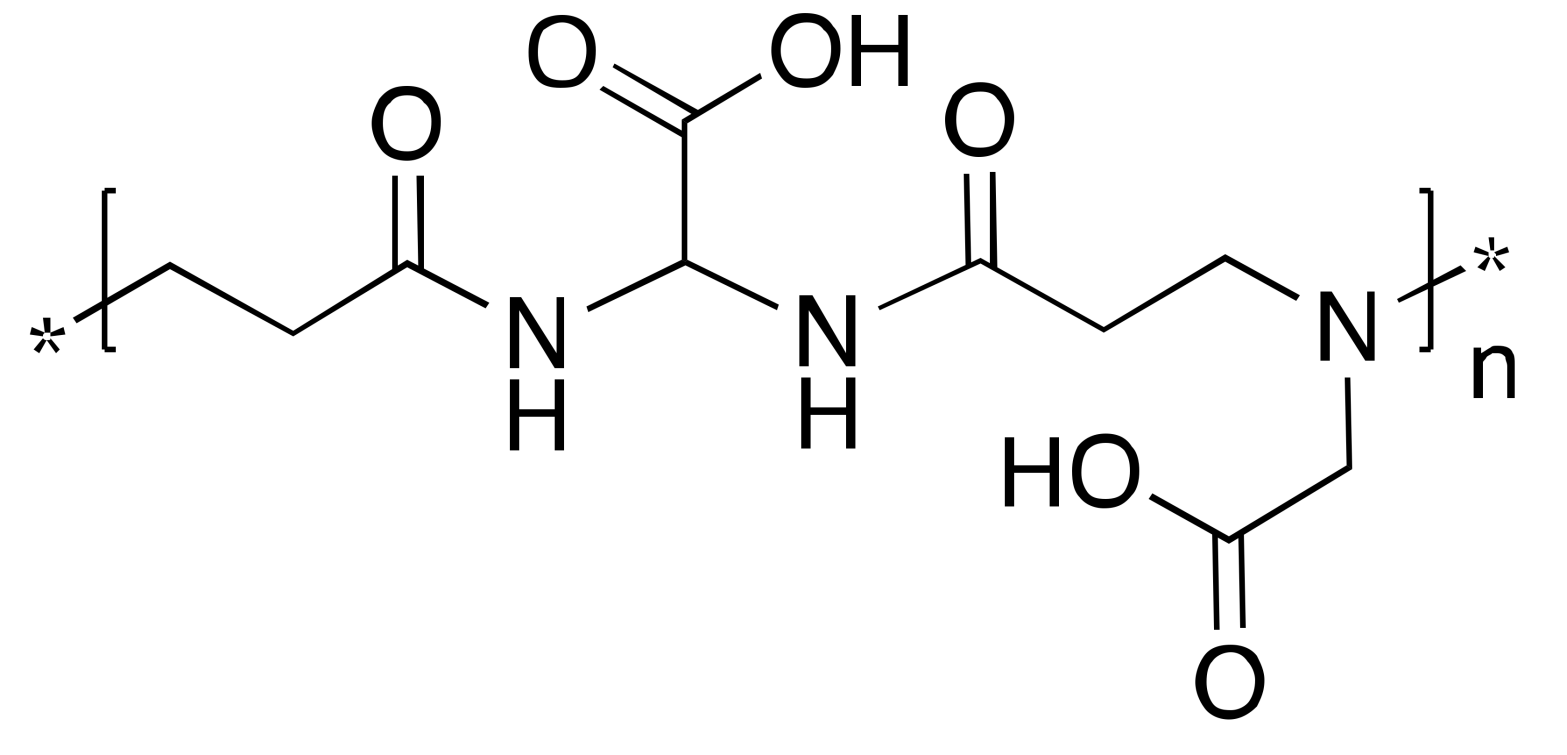	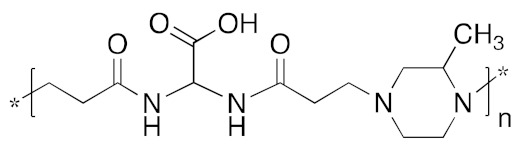
**B-EA**	**B-GLY**	**B-MP**
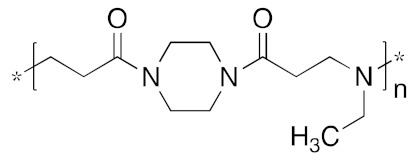	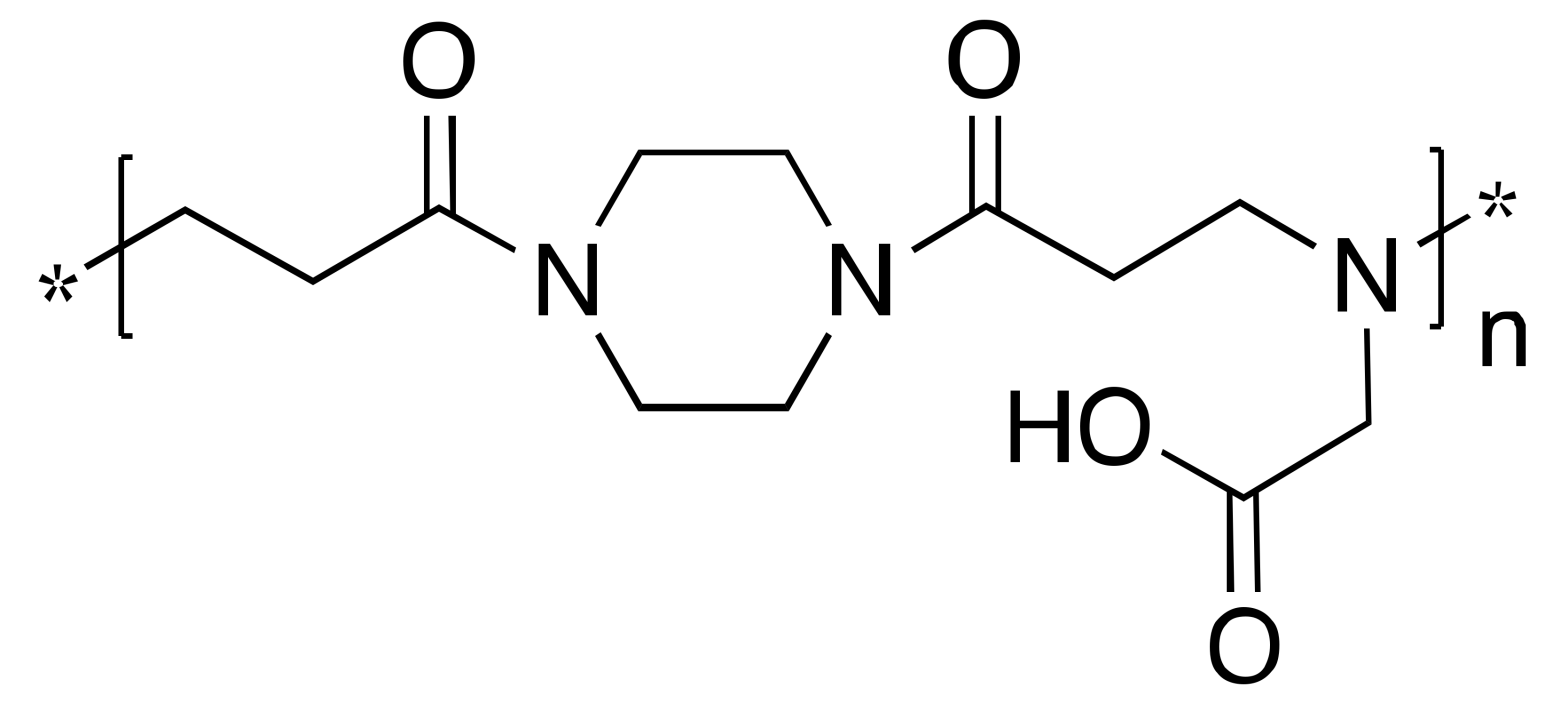	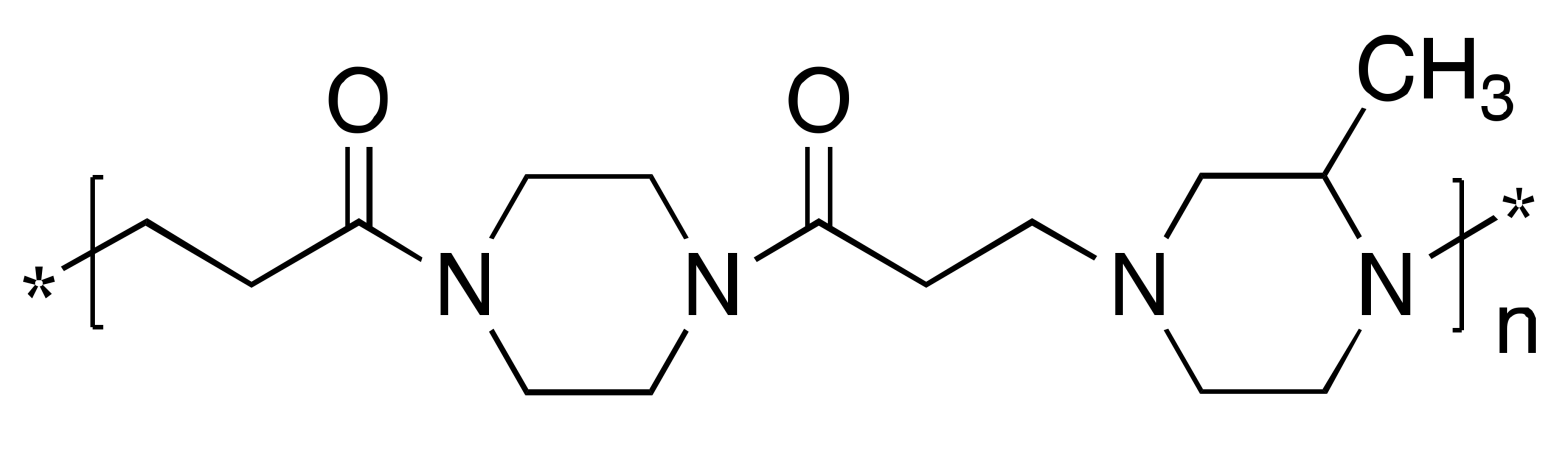
**BP-EA**	**BP-GLY**	**BP-MP**

^a^ The first part of the polymer code refers to the bisacrylamide monomer (M, B and BP); the second part refers to the amine monomer (EA, GLY and MP). The monomer structures are shown in Scheme 1.

**Table 4:** Molecular weights of PAAs.

Polymer	*M*_w_^a^	*M*_n_^b^	PD ^c^
	SEC		
M-EA	8500	3000	2.83
M-GLY	6000	5000	1.20
M-MP	7500	6000	1.25
B-EA	15,500	13,500	1.15
B-GLY	5500	5000	1.10
B-MP	34,000	26,000	1.31
BP-EA	12,500	8500	1.47
BP-GLY	6000	5000	1.20
BP-MP	19,000	17,000	1.12

^a^ Weight average molecular weights by size exclusion chromatography (SEC); ^b^ number average molecular weights by SEC. ^c^ PD = *M_w_/M_n_* (SEC).

**Table 5:** Kinetic rate constants of the retro aza-Michael reaction at 25 °C.

Sample ^a^	k_d_ (h^−1^) × 10^4 b^	
	pH 7.0	pH 9.0
M-EA	4.00	nd ^c^
M-GLY	1.00	3.50 ^d^
M-MP	0.49	nd
B-EA	2.90	5.8 ^d^
B-GLY	1.10	3.10 ^d^
B-MP	0.43	0.43
BP-EA	nd	nd
BP-GLY	nd	nd
BP-MP	0.75	0.30

^a^ Concentration of the repeat unit 43.6 μM in D_2_O. ^b^ Kinetic rate constant relative to the retro-aza-Michael reaction. The kinetic constants were calculated by fitting the experimental values of the degradation degrees obtained from the average of 2-4 determinations. ^c^ nd = not determined. ^d^ Up to 22 days (M-GLY), 30 days (B-EA) and 60 days (B-GLY).

**Table 6:** Protonation degree (α) of polymer repeat units and parent amines.

Sample	RU ^a,b^		A ^c^				α			
					pH 4.0		pH 7.0		pH 9.0	
	*pK_a1_*	*pK_a2_*	*pK_a1_*	*pK_a2_*	RU ^b^	A ^c^	RU	A	RU	A
M-EA	8.4	-	10.67 ^d^	-	1.0	1.0	0.96	1.0	0.20	0.98
M-GLY	1.9	7.7 ^e^	2.35	9.78 ^f^	1.0	1.0	0.83	1.0	0.05	0.86
M-MP	3.0	7.1	5.19	9.46 ^g^	1.1	1.94	0.56	1.1	0.01	0.74

^a^ Repeat unit. ^b^ The *pK_a_* values of the amine moieties were almost independent of the nature of the adjacent bisacrylamide moieties [53]. ^c^ Amine monomer. ^d^ Reference [54]. ^e^ Reference [19]. ^f^ Reference [55]. ^g^ Reference [56].

## Supplementary Materials

The following are available online at https://www.mdpi.com/2073-4360/12/6/1376/s1, Figure S1: FT-IR/ATR spectra of: a) M-EA, b) M-GLY and c) M-MP. Figure S2: FT-IR/ATR spectra of: a) B-EA, b) B-GLY and c) B-MP. Figure S3: FT-IR/ATR spectra of: a) BP-EA, b) BP-GLY and c) BP-MP. Figure S4: ^1^H-NMR of M-EA at pH 4.0. Figure S5: ^1^H-NMR of M-MP at pH 4.0. Figure S6: ^1^H-NMR of B-EA at pH 4.0. Figure S7: ^1^H-NMR of B-GLY at pH 4.0. Figure S8: ^1^H-NMR of B-MP at pH 4.0. Figure S9: ^1^H-NMR of BP-EA at pH 4.0. Figure S10: ^1^H-NMR of BP-GLY at pH 4.0. Figure S11: ^1^H-NMR of BP-MP at pH 4.0. Figure S12: ^1^H-NMR of M-EA at pH 7.0. Figure S13: ^1^H-NMR of M-MP at pH 7.0. Figure S14: ^1^H-NMR of B-EA at pH 7.0. Figure S15: ^1^H-NMR of B-GLY at pH 7.0. Figure S16: ^1^H-NMR of B-MP at pH 7.0. Figure S17: ^1^H-NMR of BP-EA at pH 7.0. Figure S18: ^1^H-NMR of BP-GLY at pH 7.0. Figure S19: ^1^H-NMR of BP-MP at pH 7.0. Figure S20: ^1^H-NMR of M-EA at pH 9.0. Figure S21: ^1^H-NMR of M-MP at pH 9.0. Figure S22: ^1^H-NMR of B-EA at pH 9.0. Figure S23: ^1^H-NMR of B-GLY at pH 9.0. Figure S24: ^1^H-NMR of B-MP at pH 9.0. Figure S25: ^1^H-NMR of BP-EA at pH 9.0. Figure S26: ^1^H-NMR of BP-GLY at pH 9.0. Figure S27: ^1^H-NMR of BP-MP at pH 9.0. Figure S28: ^1^H-NMR of M-GLY at pH 9.0 and 50 °C.
