# Supplementary material for: Highlight on the Mechanism of Linear Polyamidoamine Degradation in Water
Source: Polymers (Basel). 2020 Jun 19;12(6):1376. doi: 10.3390/polym12061376 (PMC7361999; doi:10.3390/polym12061376)
Supplement: Supplementary file 1 [file polymers-12-01376-s001.pdf]

Supplementary Materials

Highlight on the Mechanism of Linear Polyamidoamine Degradation in Water

Matteo Arioli, Amedea Manfredi, Jenny Alongi, Paolo Ferruti *and Elisabetta Ranucci *

[1] Dipartimento di Chimica, Università degli Studi di Milano, via C. Golgi 19, 20133 Milano, Italy; matteo.arioli@studenti.unimi.it (M.A.); amedea.manfredi@unimi.it (A.M.); jenny.alongi@unimi.it (J.A.)

* Correspondence: paolo.ferruti@unimi.it (P.F.); elisabetta.ranucci@unimi.it (E.R.); Tel.: +39–02-50314128 (P.F.); +39–02-50314132 (E.R.)

Pages S1-S29

Figures S1-S3: FT-IR/ATR spectra of PAAs.

Figures S4-S27: ^{1}H-NMR spectra of PAAs at pH 4.0, 7.0 and 9.0.

Figure S28: ^{1}H-NMR spectra of M-GLY at pH 9.0 and 50 °C.

FT-IR/ATR characterization

All PAAs were analyzed by attenuated total reflectance (ATR) Fourier transform infrared spectroscopy (FT-IR). FT-IR/ATR spectra were recorded at room temperature, in the 4000 - 380 cm^{-1} wavenumber range, with 32 scans and 4 cm^{-1} resolution using a Perkin-Elmer Frontier FT-IR/FIR spectrophotometer (Milano, Italy), equipped with a diamond crystal characterized by a penetration depth of 1.66 μm.

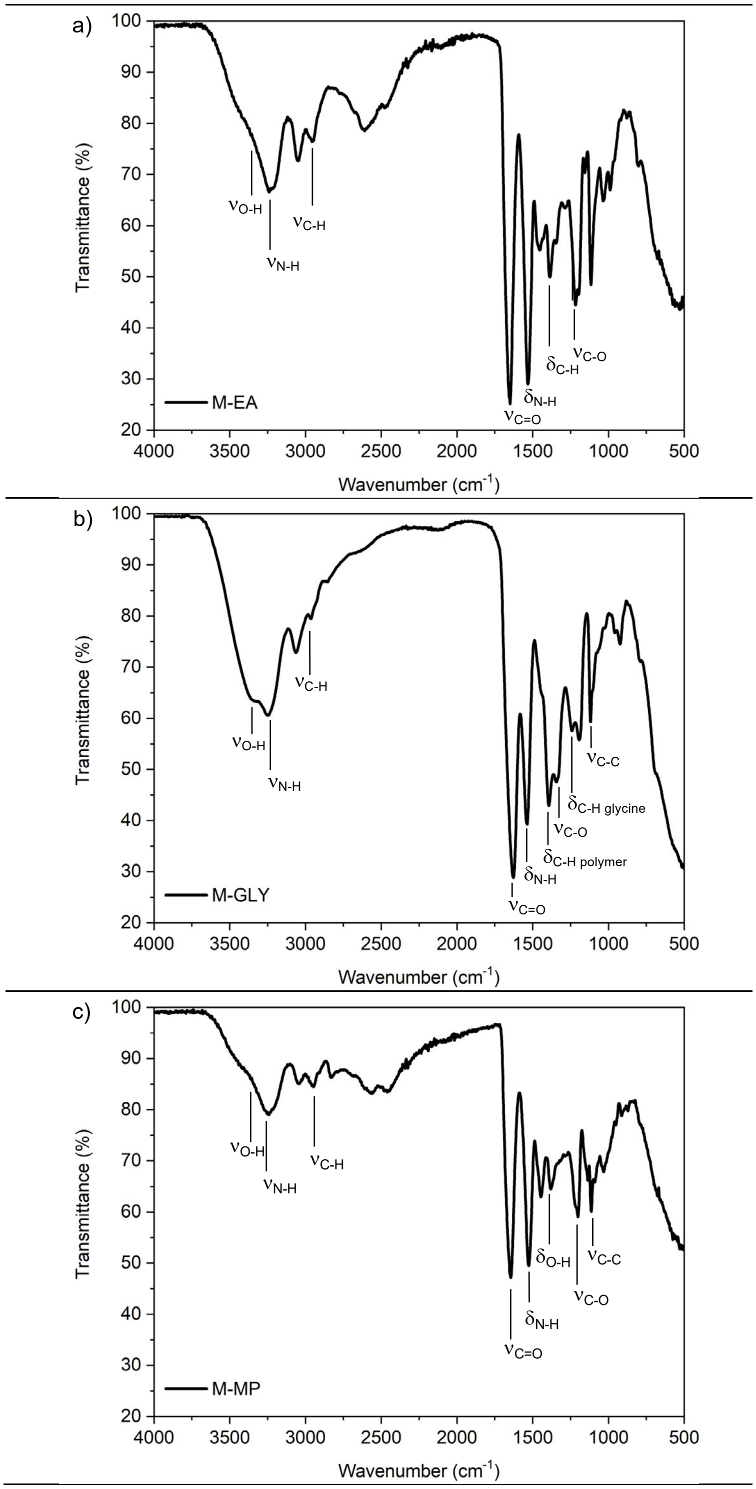

Figure S1. FT-IR/ATR spectra of: a) M-EA, b) M-GLY and c) M-MP.

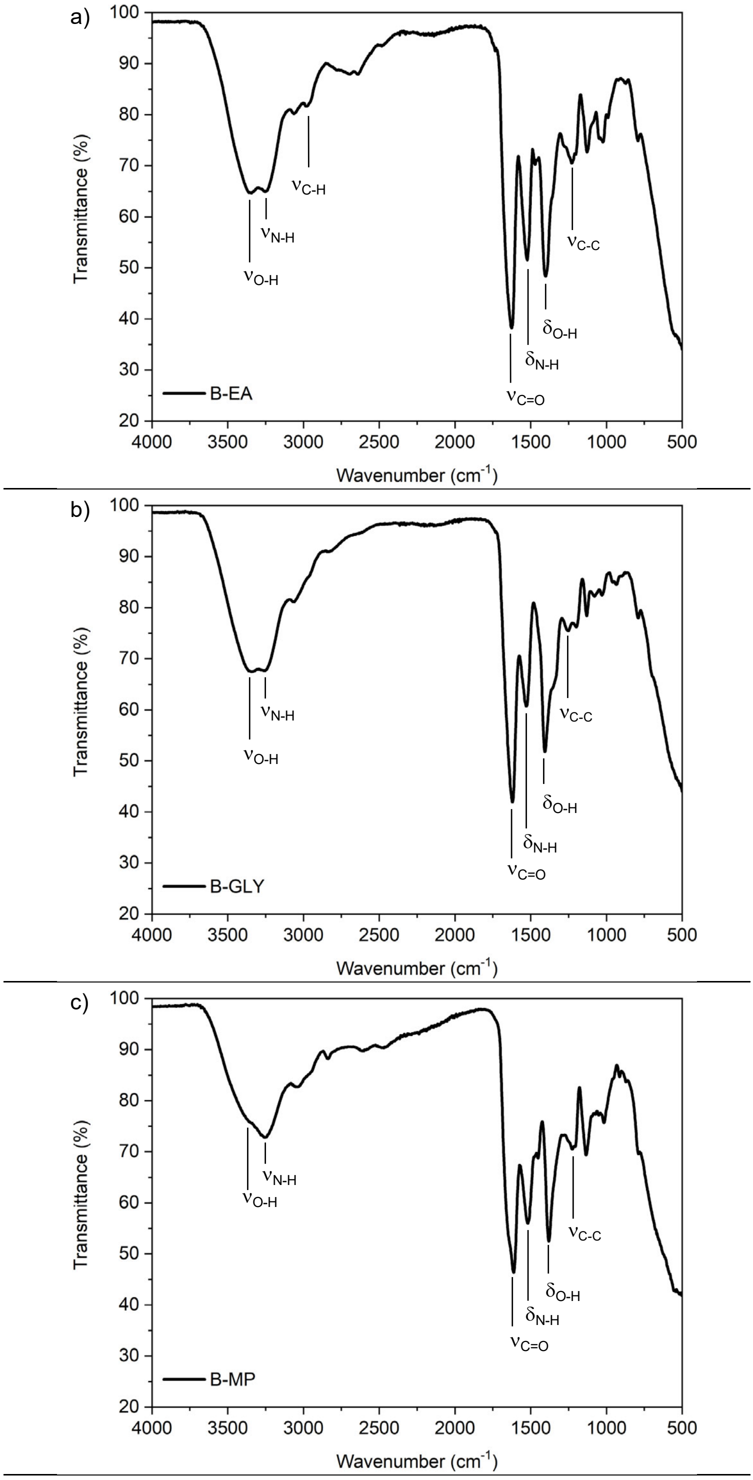

Figure S2. FT-IR/ATR spectra of: a) B-EA, b) B-GLY and c) B-MP.

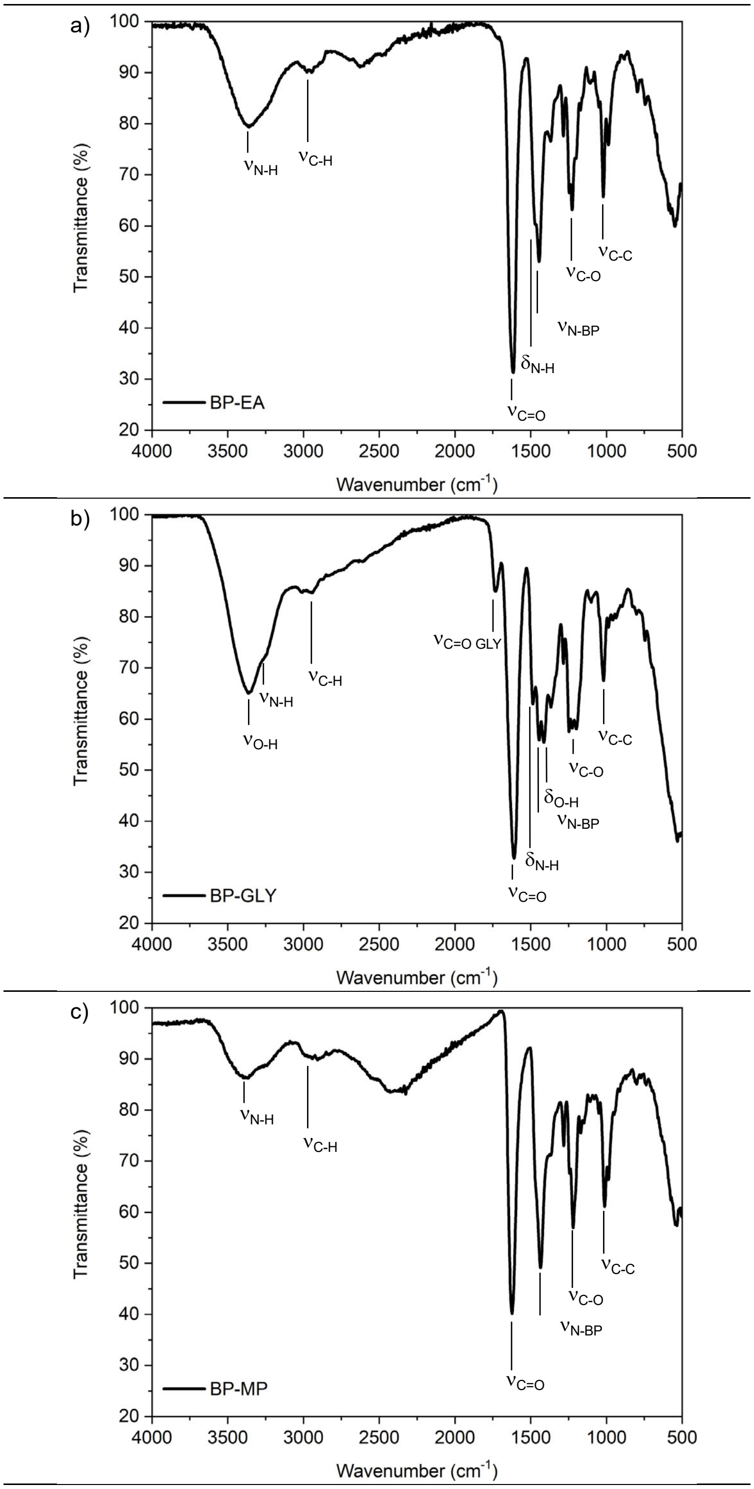

Figure S3. FT-IR/ATR spectra of: a) BP-EA, b) BP-GLY and c) BP-MP.

^{1}H-NMR characterization

All PAAs were characterized by ^{1}H-NMR spectroscopy, using a Brüker Avance DPX-400 NMR spectrometer (Milano, Italy) operating at 400.13 MHz. Number of scans 32, relaxation delay, *d1*, 10.0 s, receiver gain automatically measured and set by the instrument. Analyses were conducted in D_2O, adjusting the pH with D_2O solutions of DCl or NaOD.

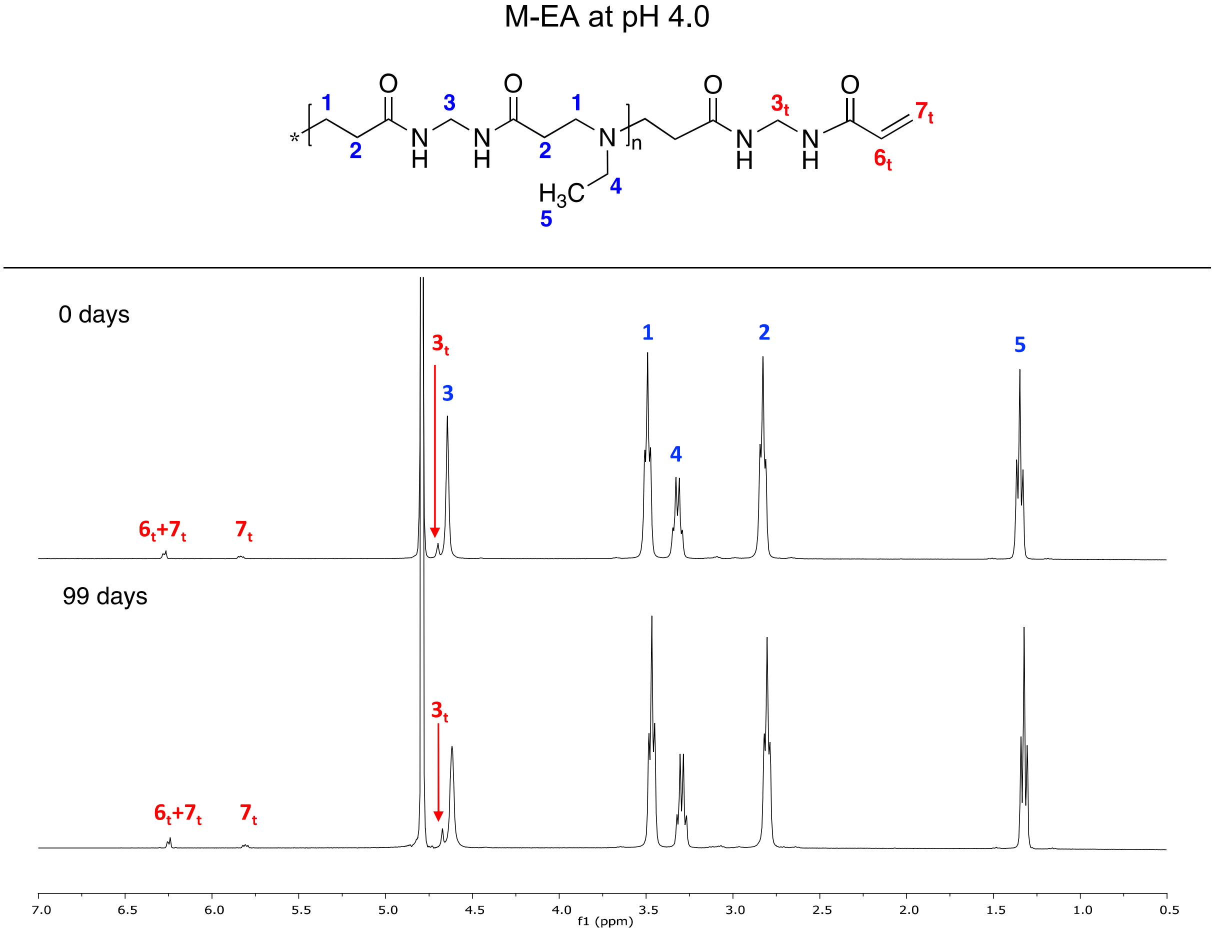

Figure S4. ^{1}H-NMR of M-EA at pH 4.0.

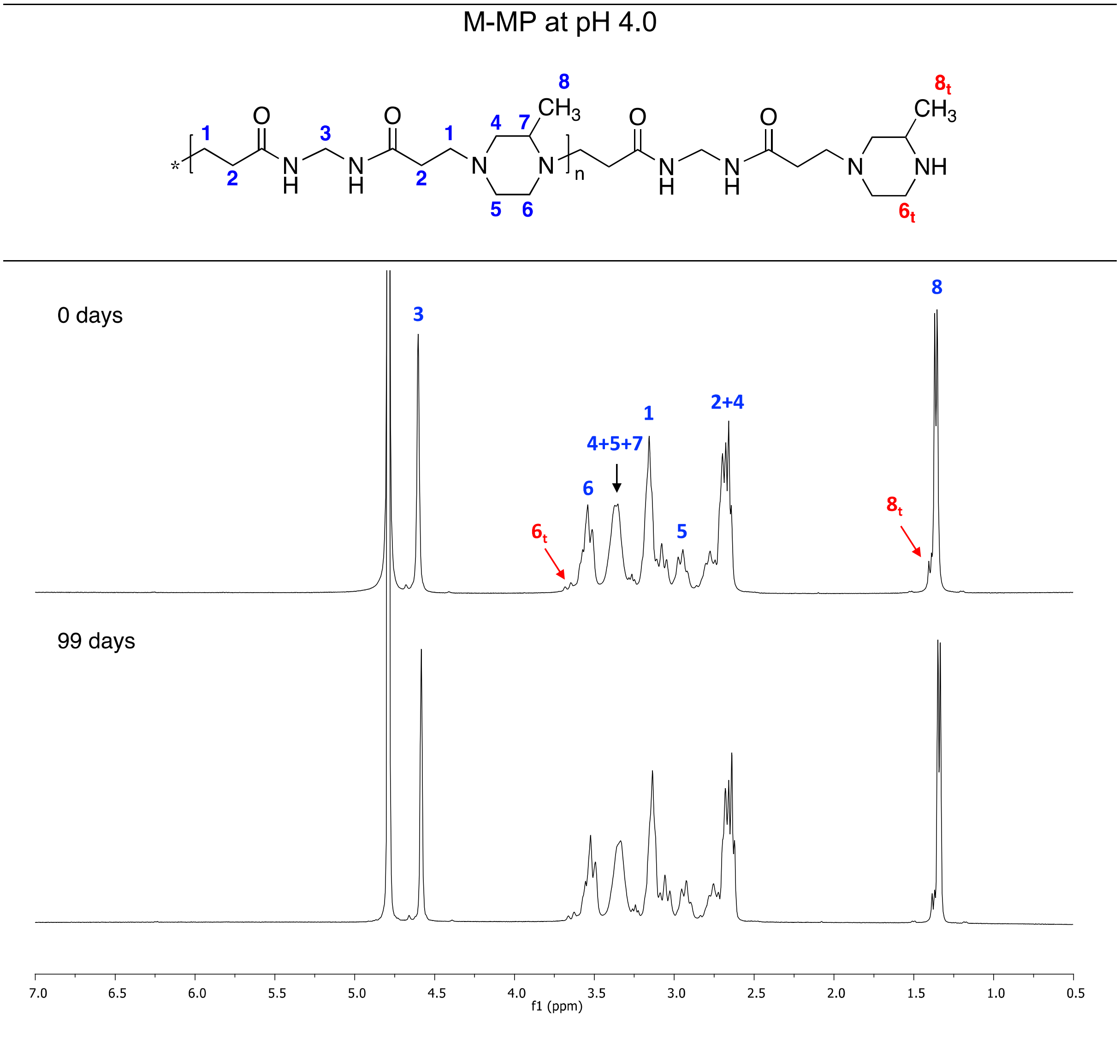

Figure S5. ^{1}H-NMR of M-MP at pH 4.0.

B-EA at pH 4.0

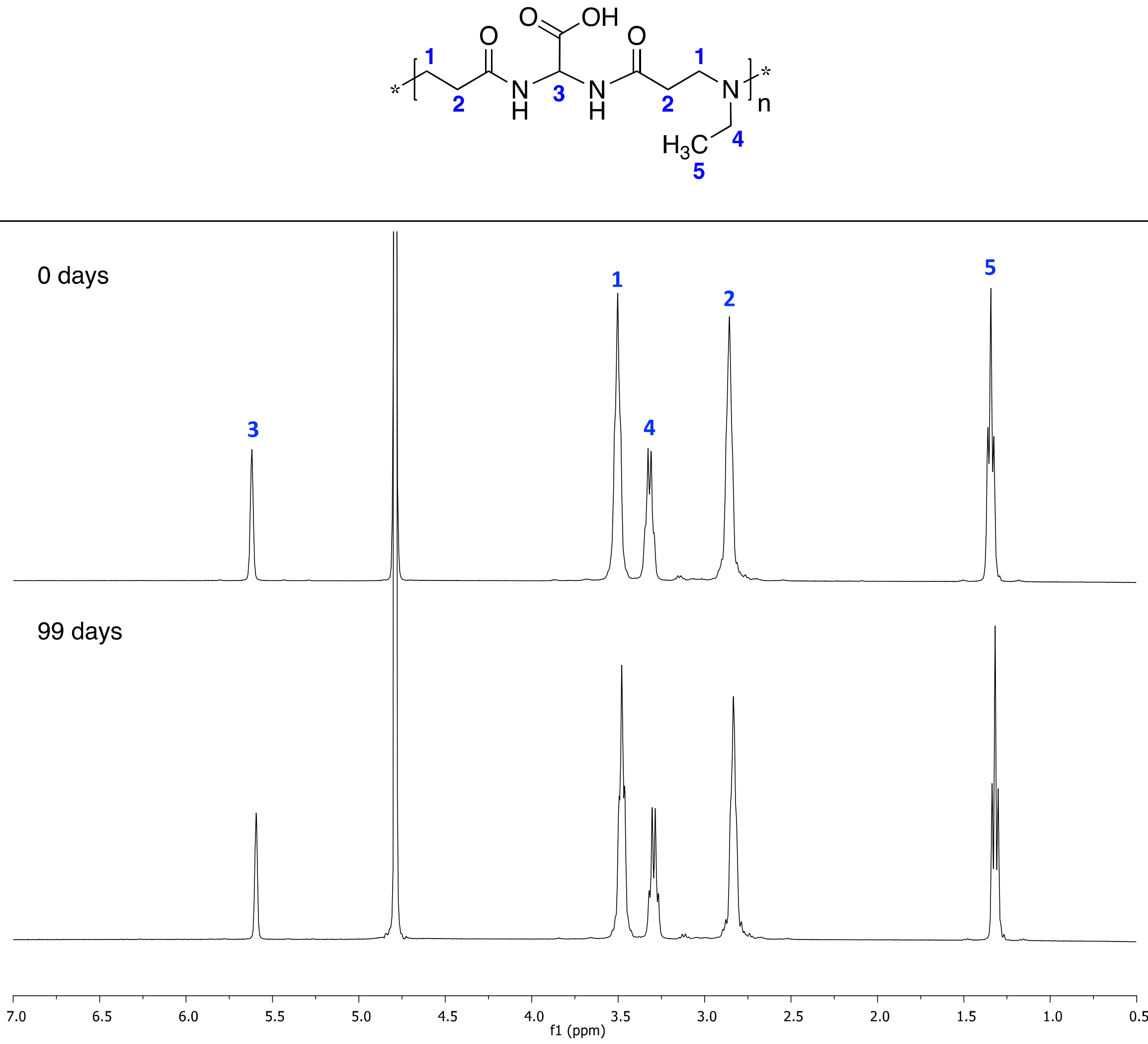

Figure S6. ^{1}H-NMR of B-EA at pH 4.0.

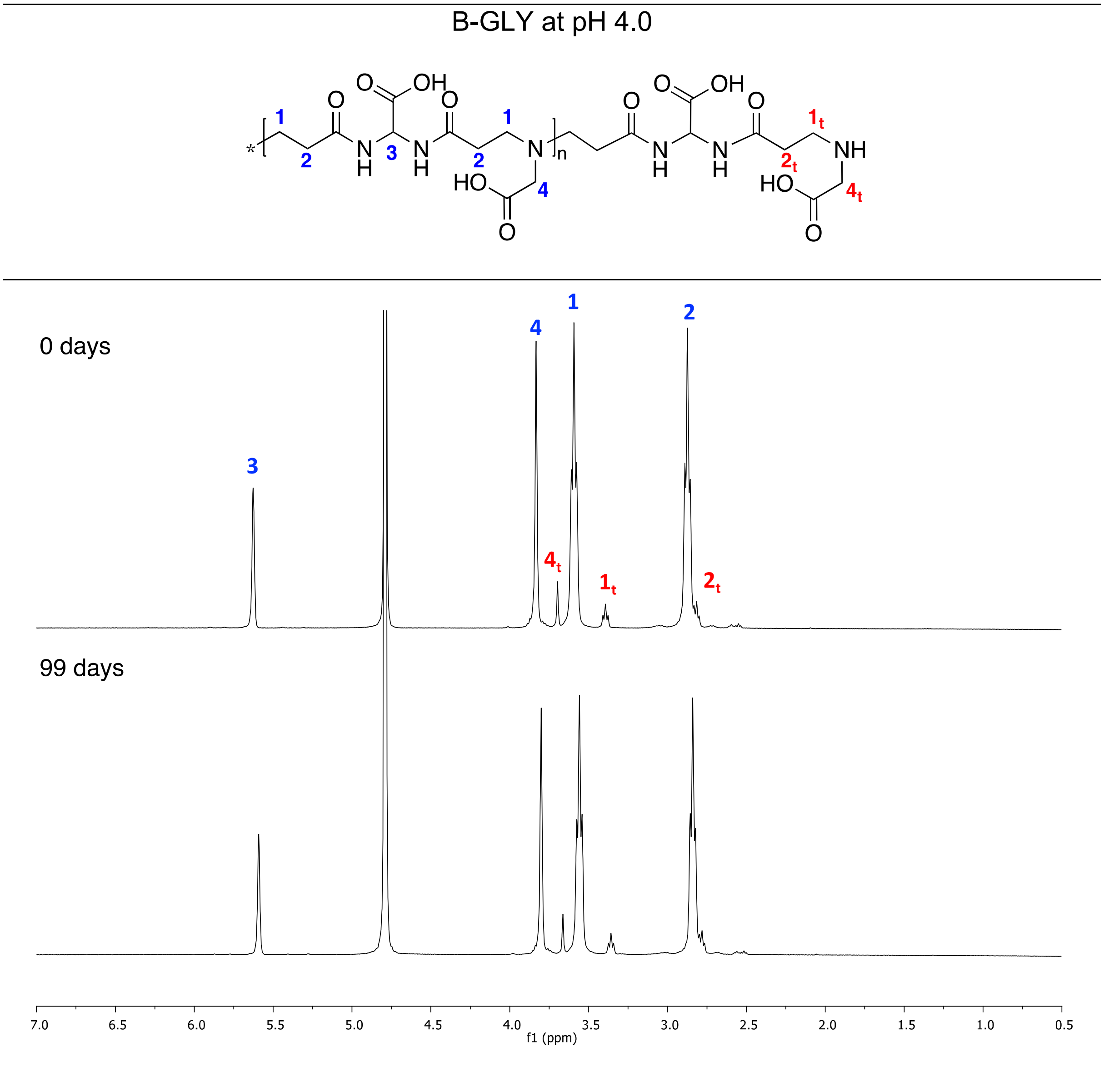

Figure S7. ^{1}H-NMR of B-GLY at pH 4.0.

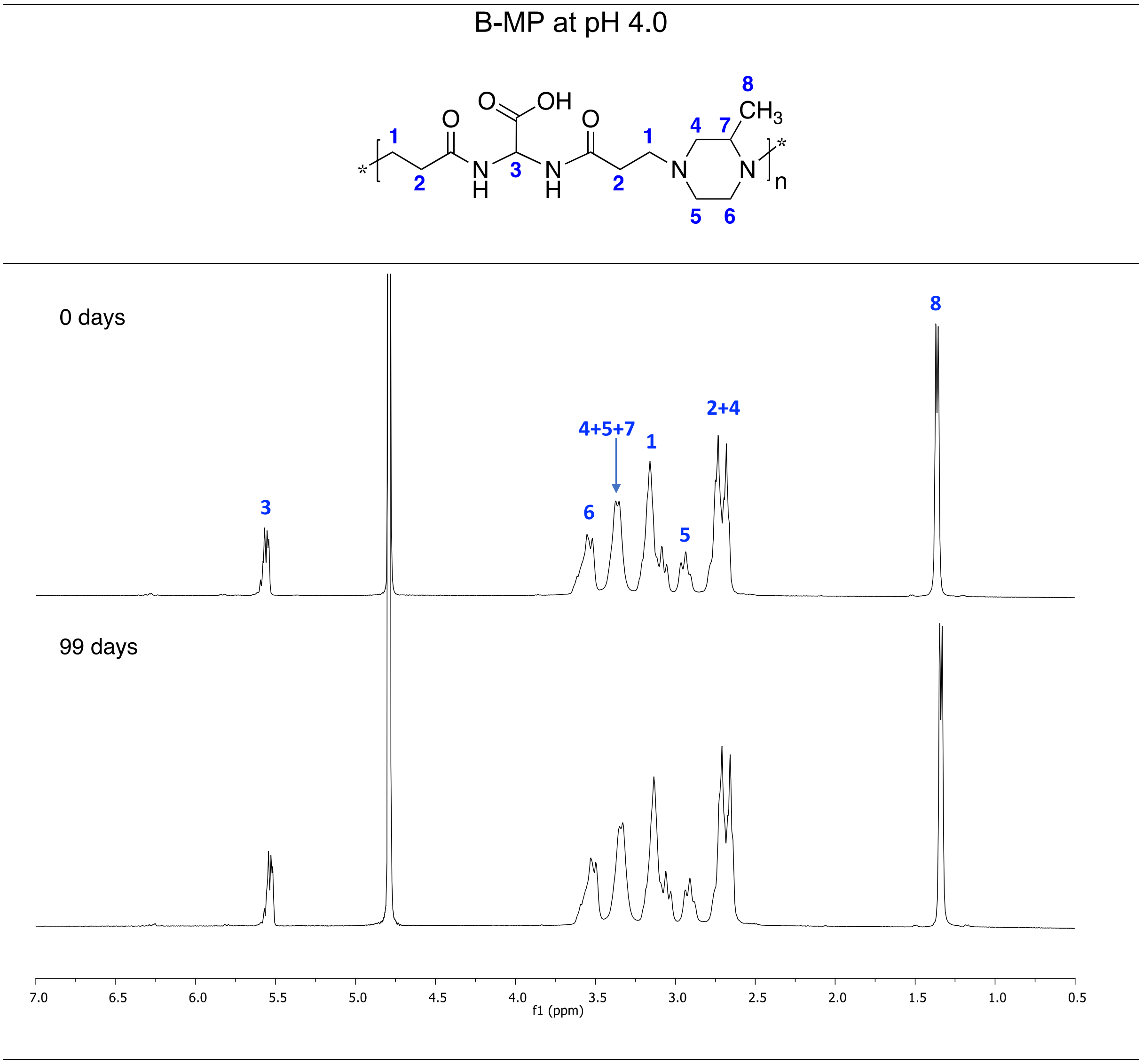

Figure S8. ^{1}H-NMR of B-MP at pH 4.0.

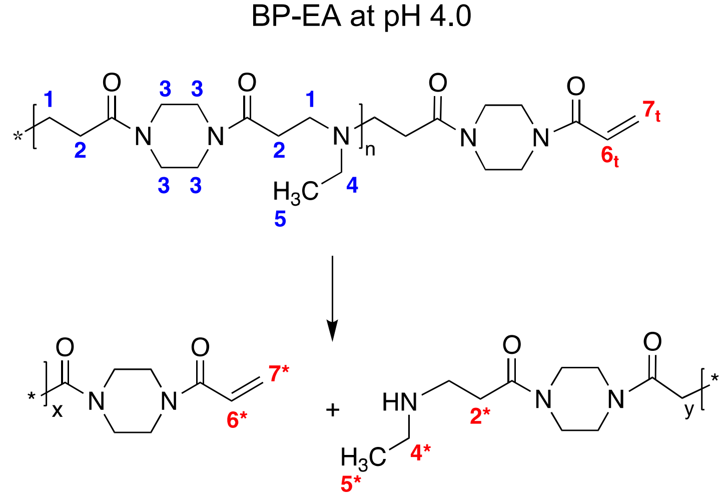

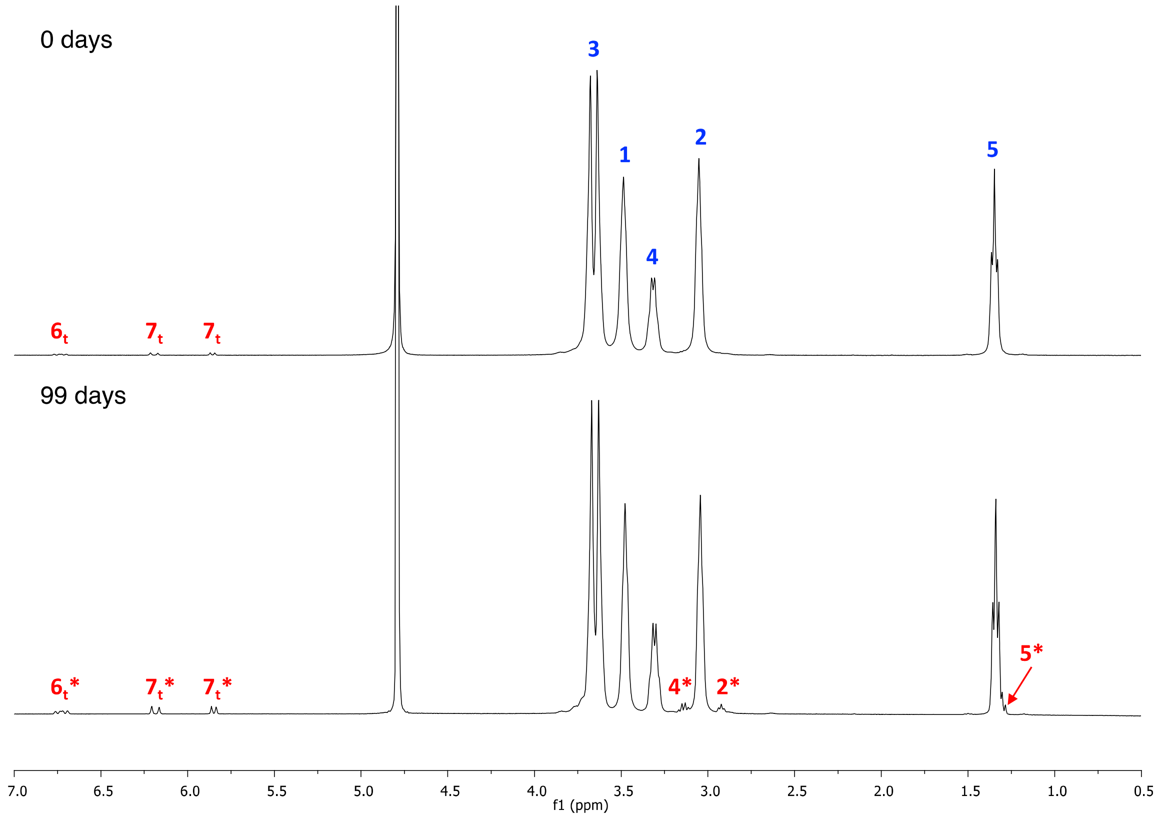

Figure S9. ^{1}H-NMR of BP-EA at pH 4.0.

BP-GLY at pH 4.0

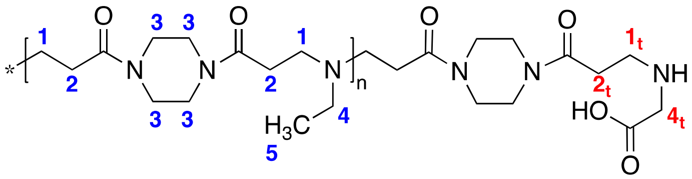

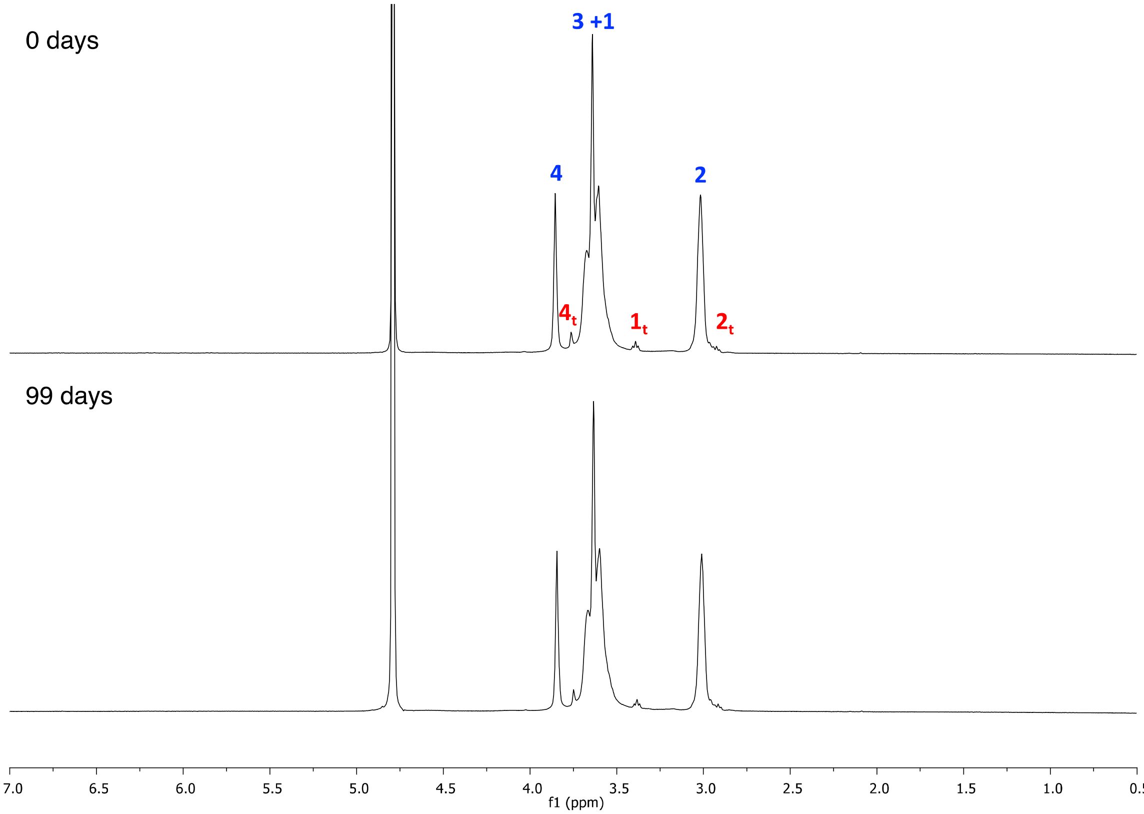

Figure S10. ^{1}H-NMR of BP-GLY at pH 4.0.

BP-MP at pH 4.0

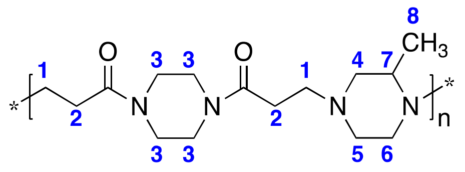

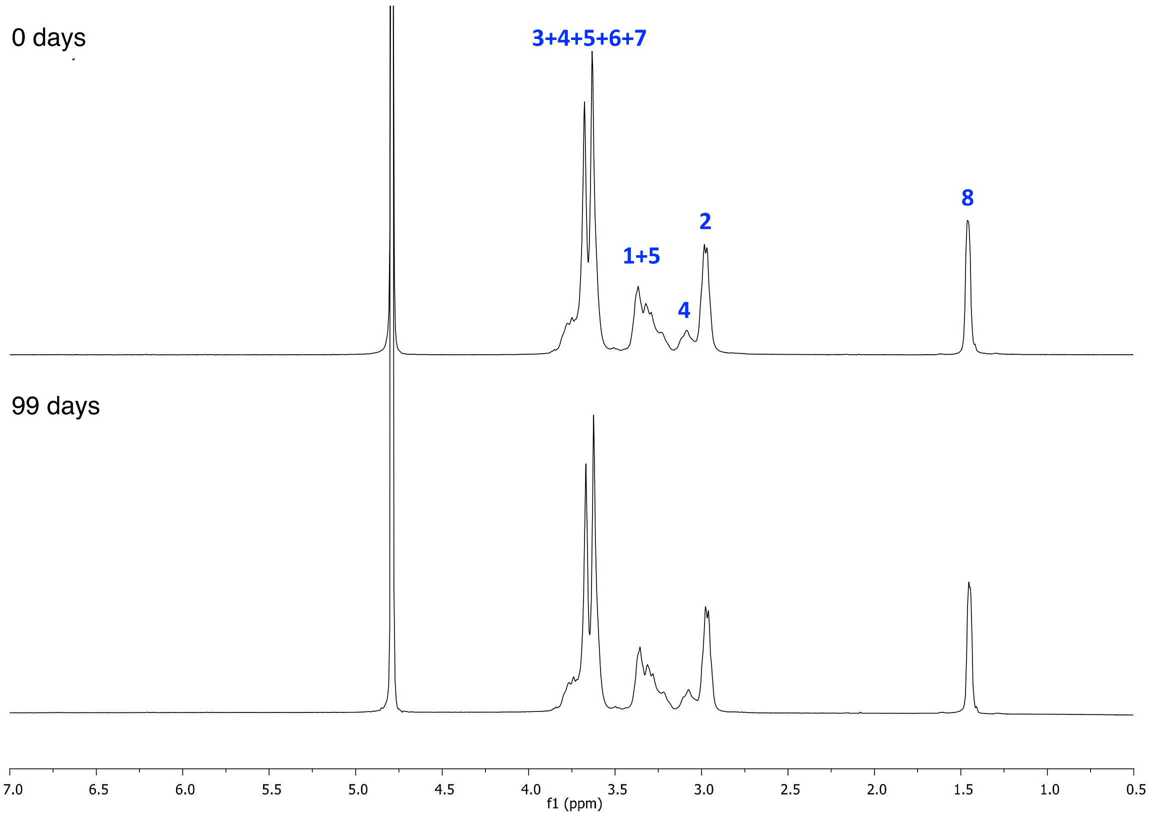

Figure S11. ^{1}H-NMR of BP-MP at pH 4.0.

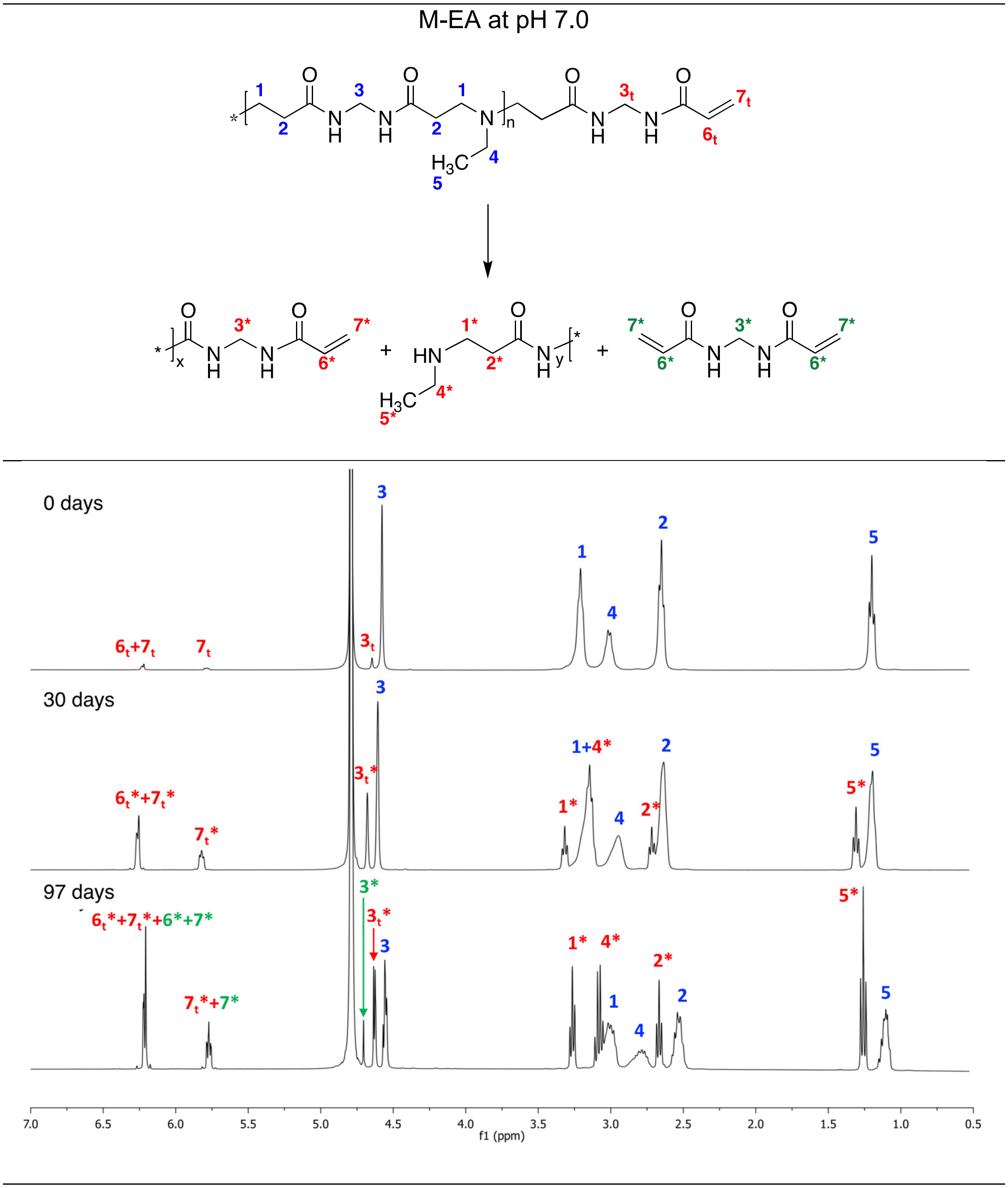

Figure S12. ^{1}H-NMR of M-EA at pH 7.0.

Degradation percent calculated both as

$[H_{3^*}/(H_3+H_{3^*})]\ x\ 100$ and

$[H_{5^*}/(H_5+H_{5^*})]\ x\ 100$ and

$\{[(H_{6^*}+H_{7^*})/3]/[(H_2+2H_{2^*})/4]\}\ x\ 100$

where H_n stands for the integral of the resonance peak of the hydrogen indicated in the subscript.

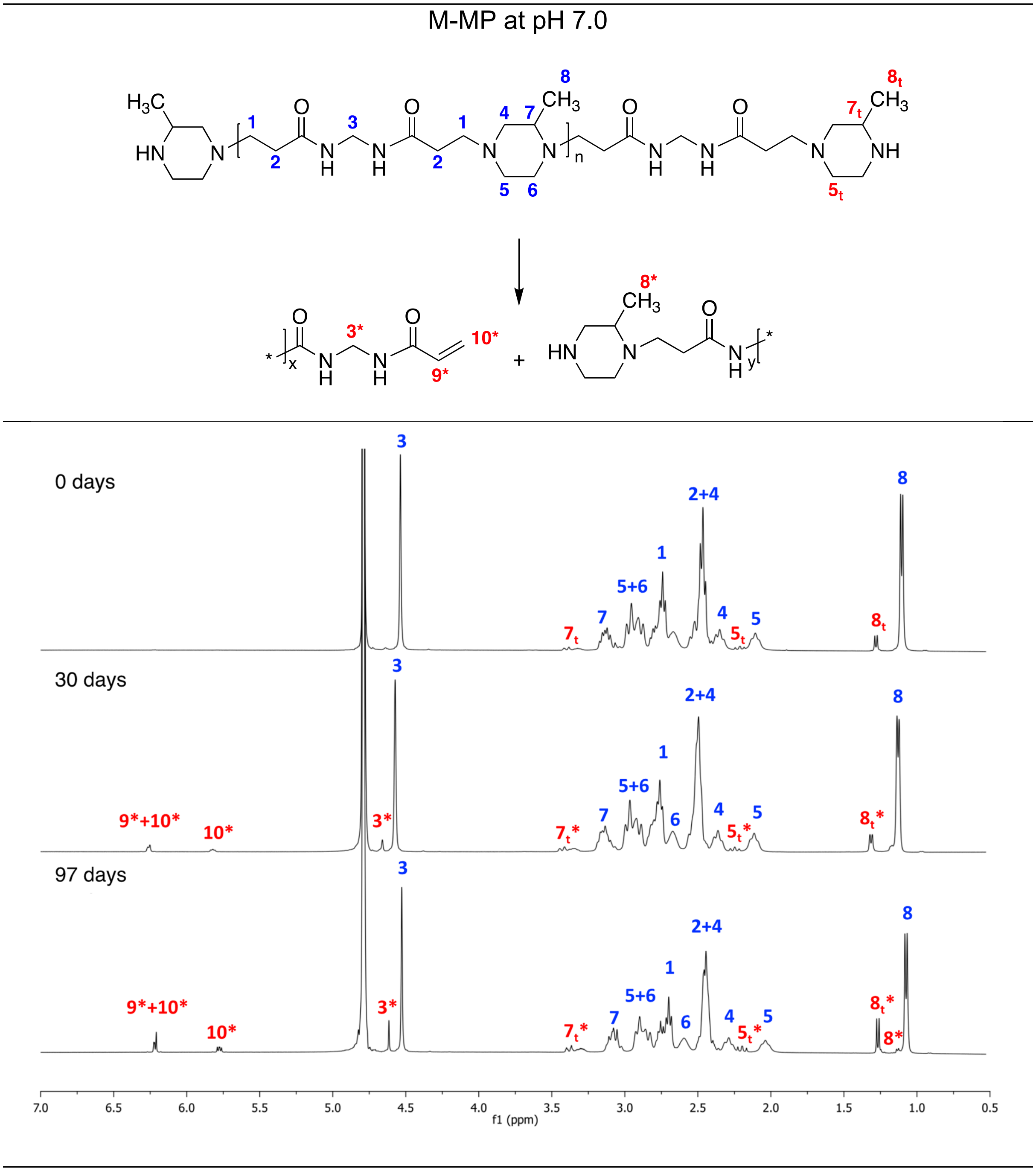

Figure S13. ^{1}H-NMR of M-MP at pH 7.0.

Degradation percent calculated both as

$[H_{3*}/(H_3+H_{3*})] \times 100$ and

$[H_{8*}/(H_8+H_{8*})] \times 100$ and

$\{[(H_{9*}+H_{10*})/3]/H_7\} \times 100$

where H_n stands for the integral of the resonance peak of the hydrogen indicated in the subscript.

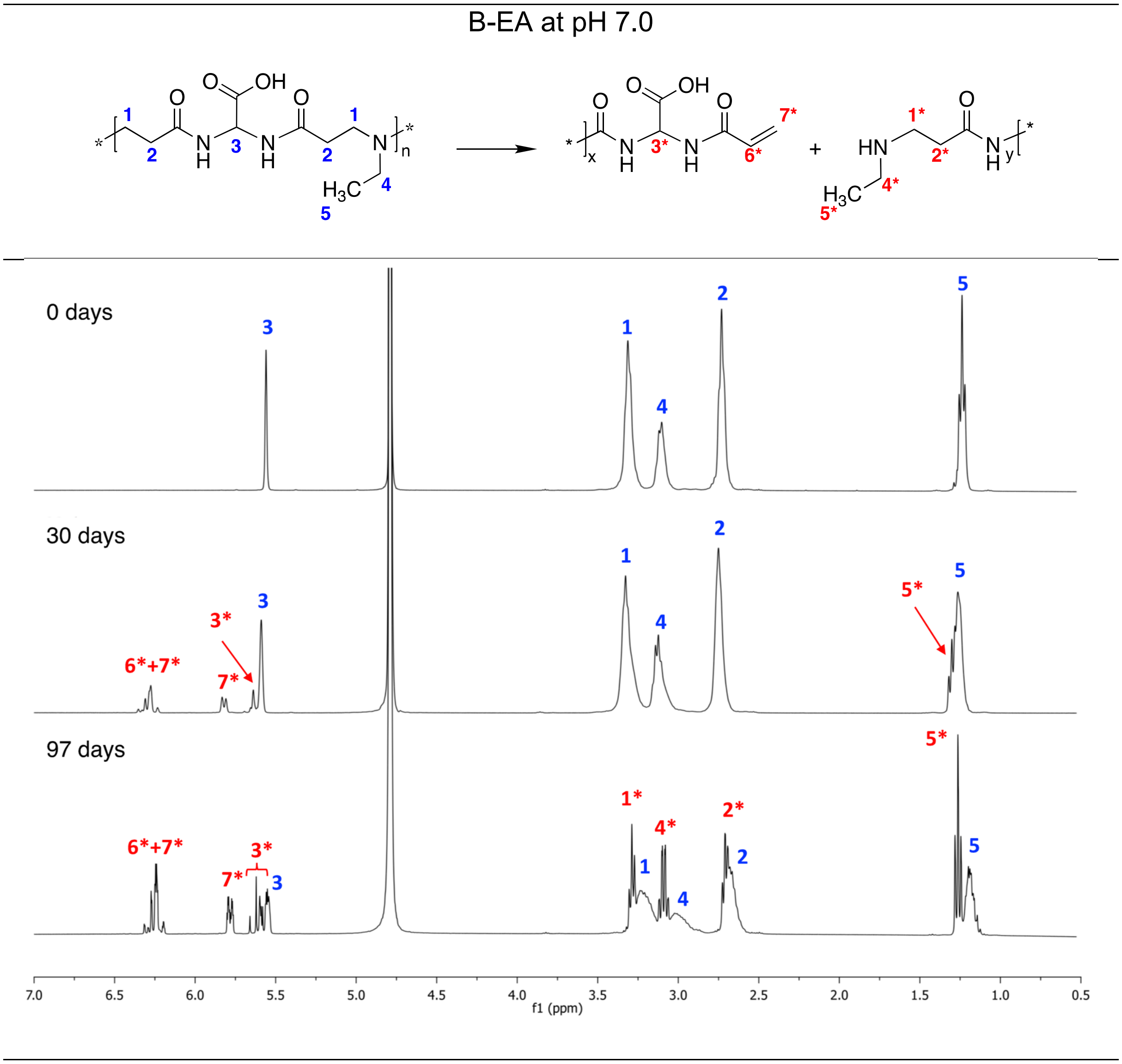

Figure S14. ^{1}H-NMR of B-EA at pH 7.0.

Degradation percent calculated both as

$[H_{3^*}/(H_3+H_{3^*})]$ *x 100* and

$[(H_{6^*}+H_{7^*})/(H_5+H_{5^*})]$ *x 100* and

$\{[(H_{6^*}+H_{7^*})/3/(H_3+H_{3^*})]\}$ *x 100*

where H_n stands for the integral of the resonance peak of the hydrogen indicated in the subscript.

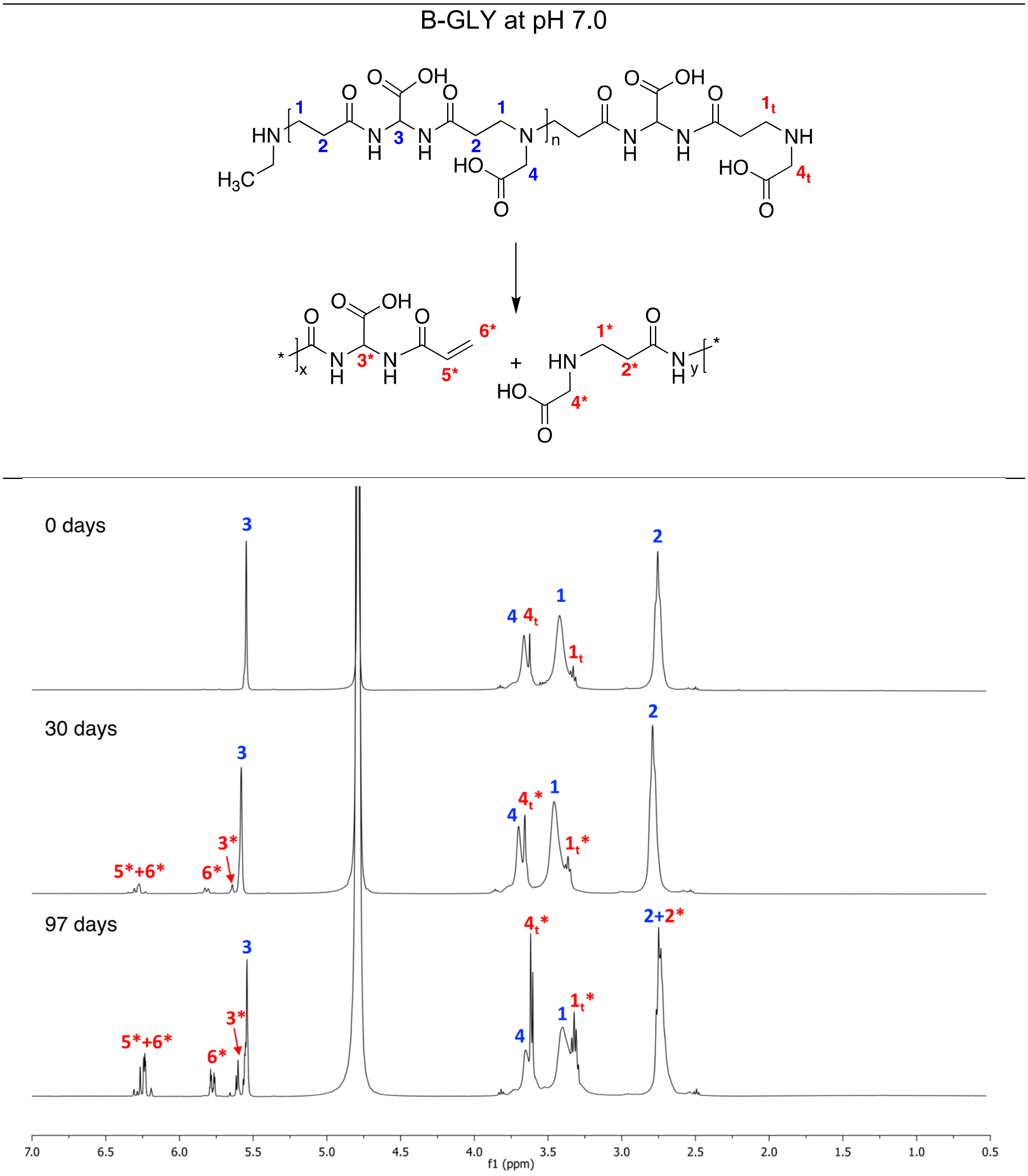

Figure S15. ^{1}H-NMR of B-GLY at pH 7.0.

Degradation percent calculated both as

$[H_{3^*}/(H_3+H_{3^*})] \times 100$ and

$\{[(H_{5^*}+H_{6^*})/3/(H_3+H_{3^*})]\} \times 100$

where H_n stands for the integral of the resonance peak of the hydrogen indicated in the subscript.

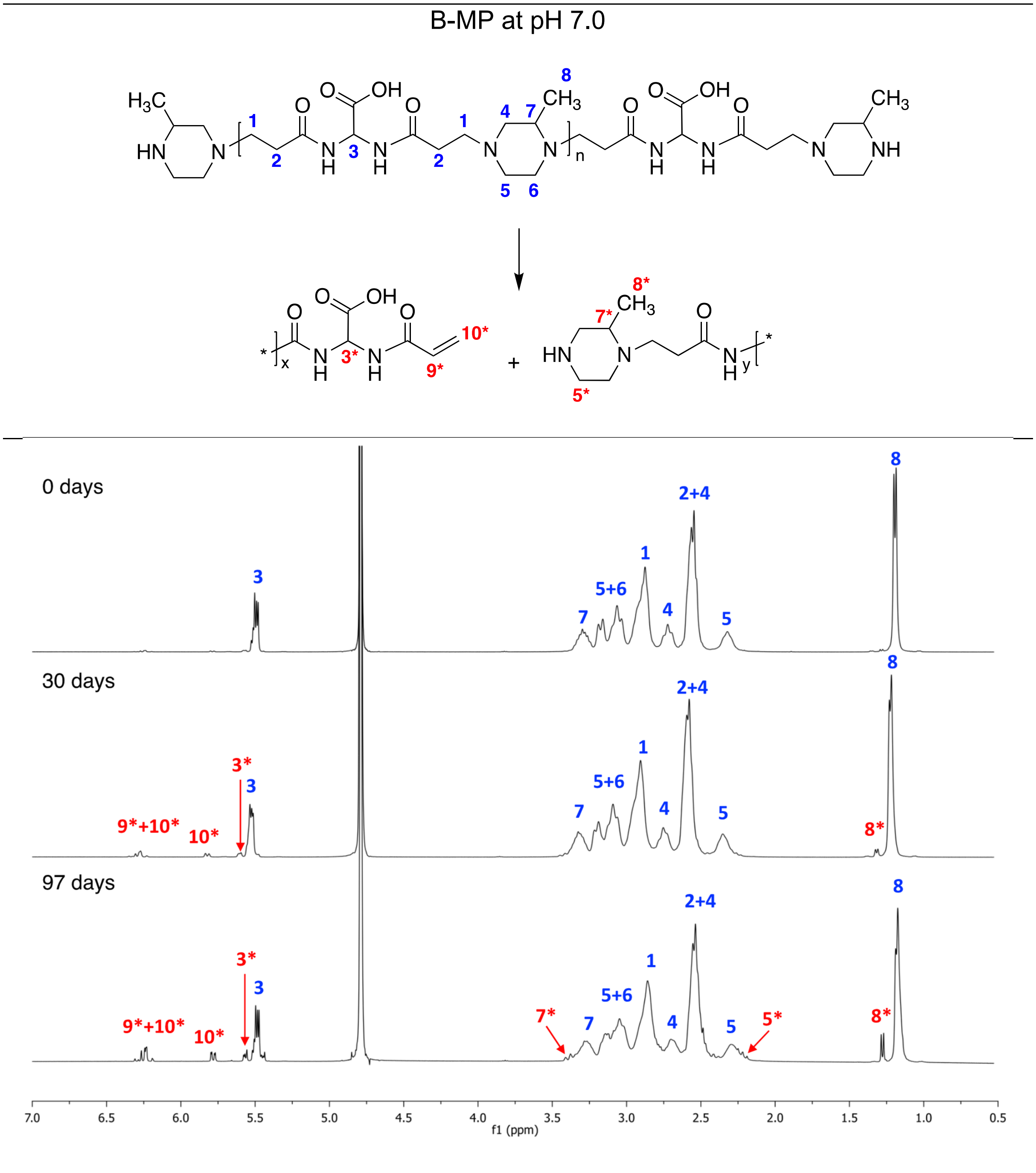

Figure S16. ^{1}H-NMR of B-MP at pH 7.0.

Degradation percent calculated both as

$[H_{3^*}/(H_3+H_{3^*})] \times 100$ and

$[H_{8^*}/(H_8+H_{8^*})] \times 100$ and

$\{[(H_{9^*}+H_{10^*})/3/(H_7+H_{7^*})]\} \times 100$

where H_n stands for the integral of the resonance peak of the hydrogen indicated in the subscript.

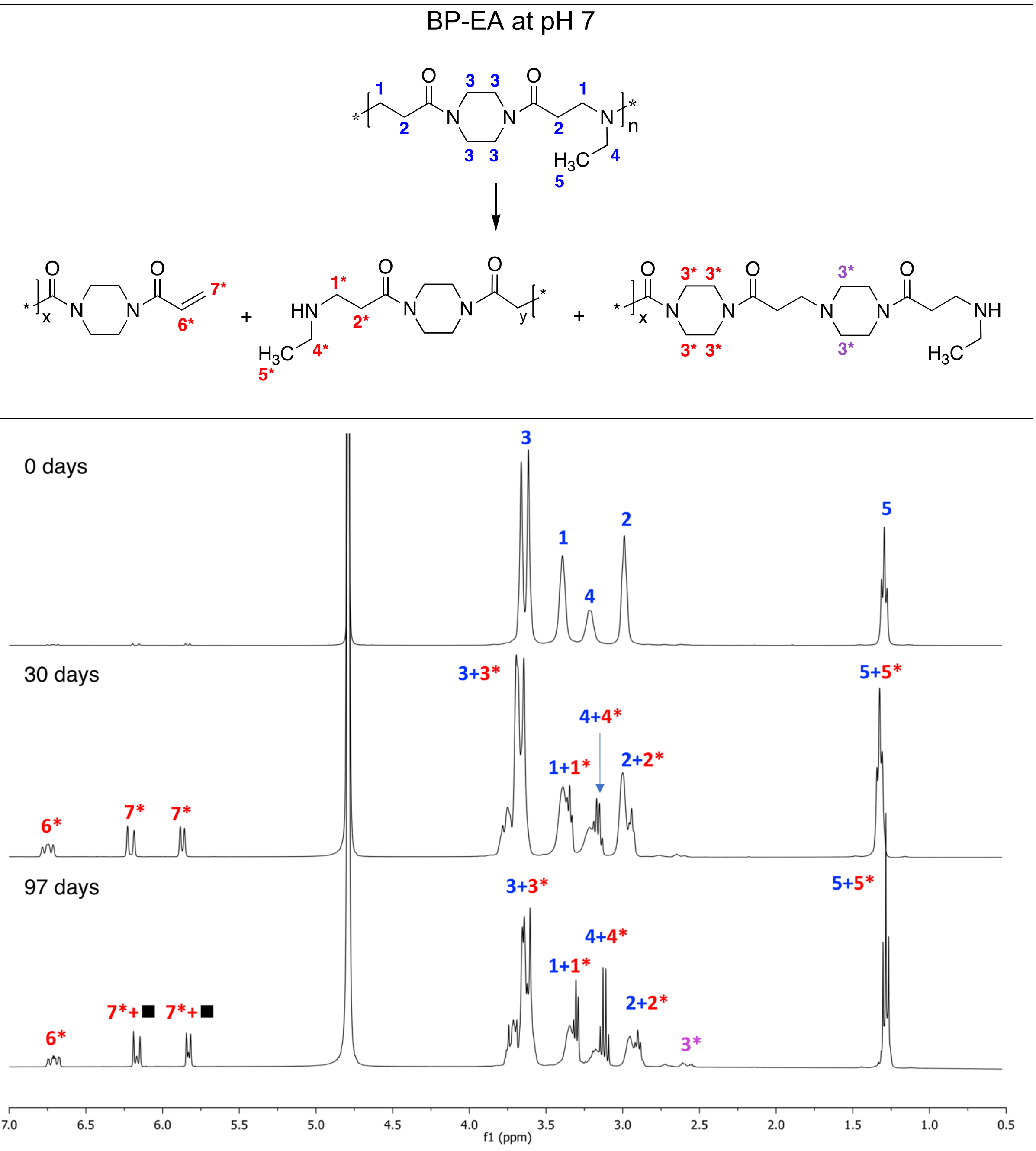

Figure S17. ^{1}H-NMR of BP-EA at pH 7.0.

■ Side products.

Degradation percent calculated both as

$\{[(H_{6^*}+H_{7^*})/(H_5+H_{5^*})]\}\ x\ 100$ and

$\{[(H_{6^*}+H_{7^*})/3]/[(H_3+H_{3^*})/8]\}\ x\ 100$

where H_n stands for the integral of the resonance peak of the hydrogen indicated in the subscript.

BP-GLY at pH 7.0

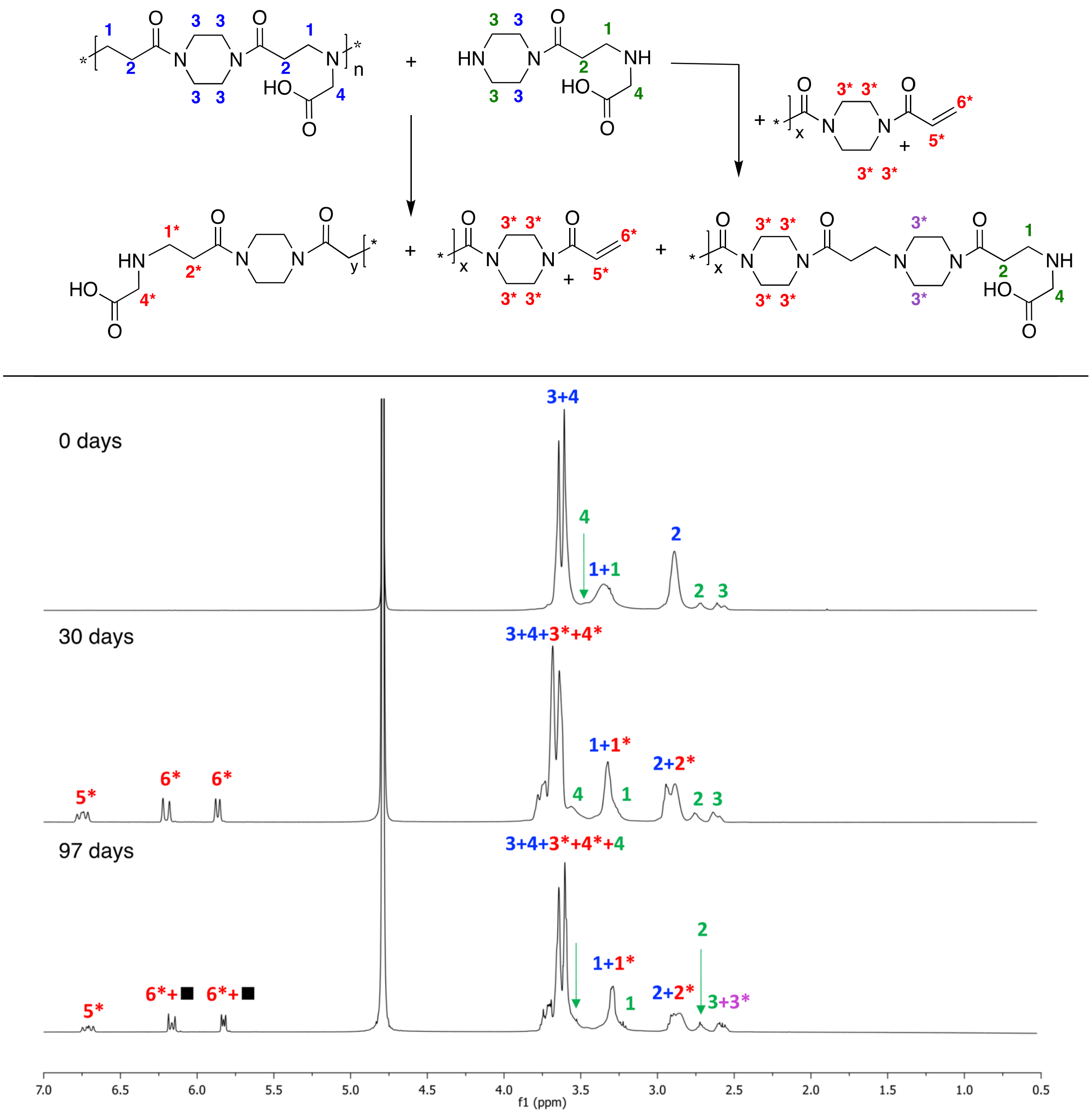

Figure S18. ^{1}H-NMR of BP-GLY at pH 7.0.

■ Side products.

Degradation percent calculated as

$\{[(H_{5^*}+H_{6^*})/3]/[(H_3+H_4+H_{3^*}+H_{4^*})/10]\} \times 100$

where H_n stands for the integral of the resonance peak of the hydrogen indicated in the subscript.

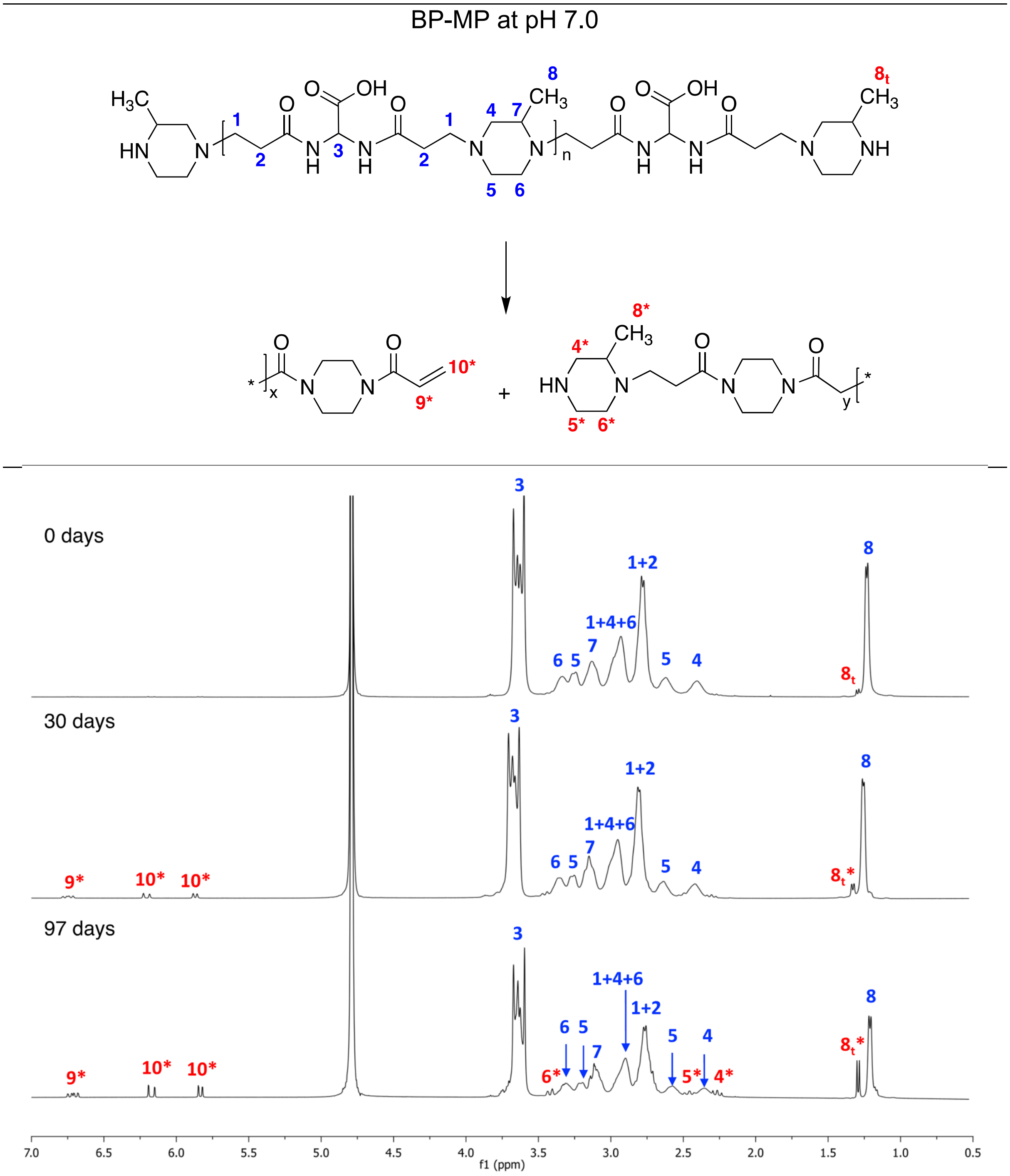

Figure S19. ^{1}H-NMR of BP-MP at pH 7.0.

Degradation percent calculated both as

$[H_{8^*}/(H_8+H_{8^*})] \times 100$ and

$\{[(H_{9^*}+H_{10^*})/3]/[(H_3)/8]\} \times 100$

where H_n stands for the integral of the resonance peak of the hydrogen indicated in the subscript.

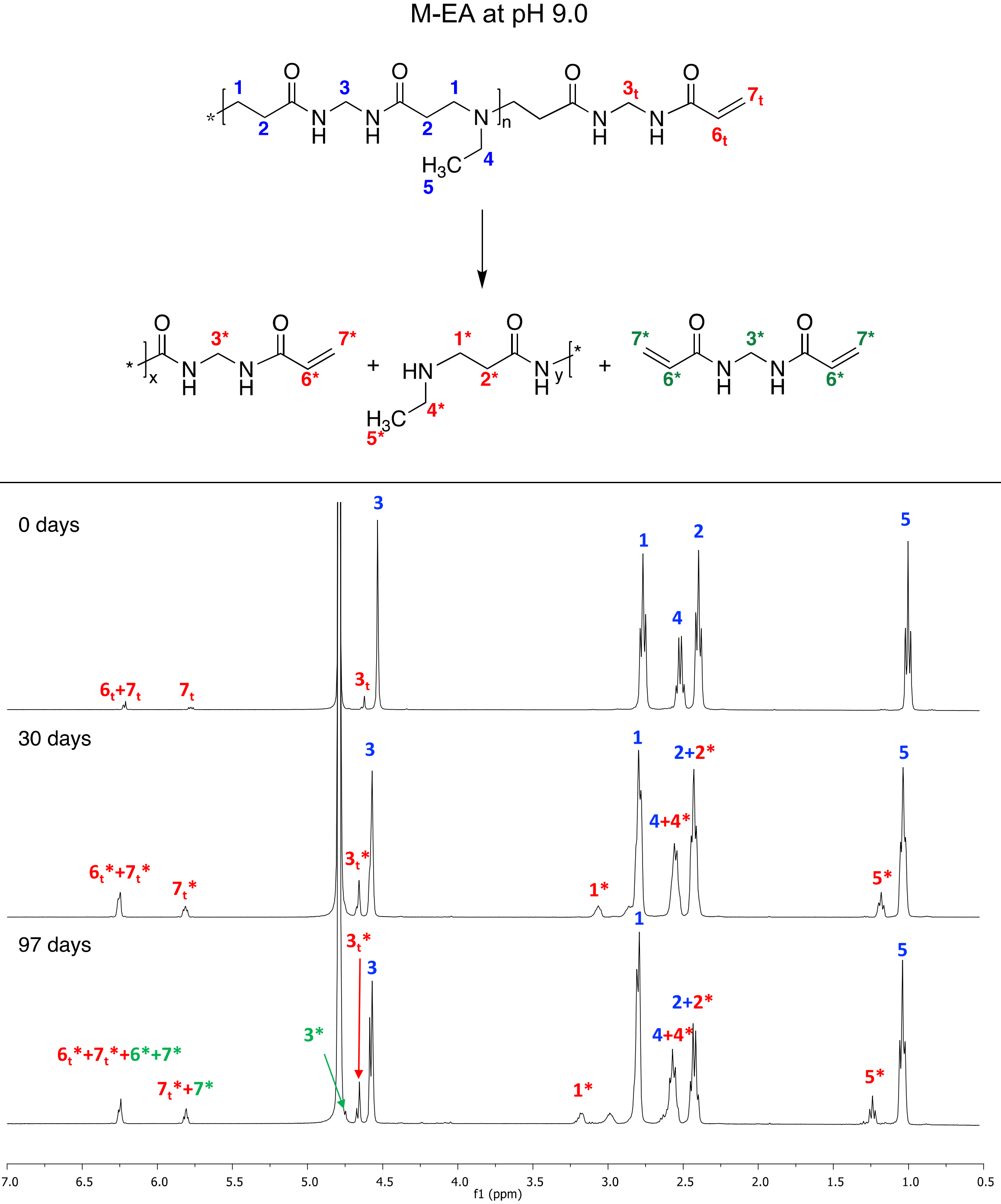

Figure S20. ^{1}H-NMR of M-EA at pH 9.0.

Degradation percent calculated both as

$[H_{3^*}/(H_3+H_{3^*})] \times 100$ and

$[H_{5^*}/(H_5+H_{5^*})] \times 100$ and

$\{[(H_{6^*}+H_{7^*})/3]/[(H_1+H_2+H_4+H_{4^*}+2H_{1^*}+2H_{2^*})/4]\} \times 100$

where H_n stands for the integral of the resonance peak of the hydrogen indicated in the subscript.

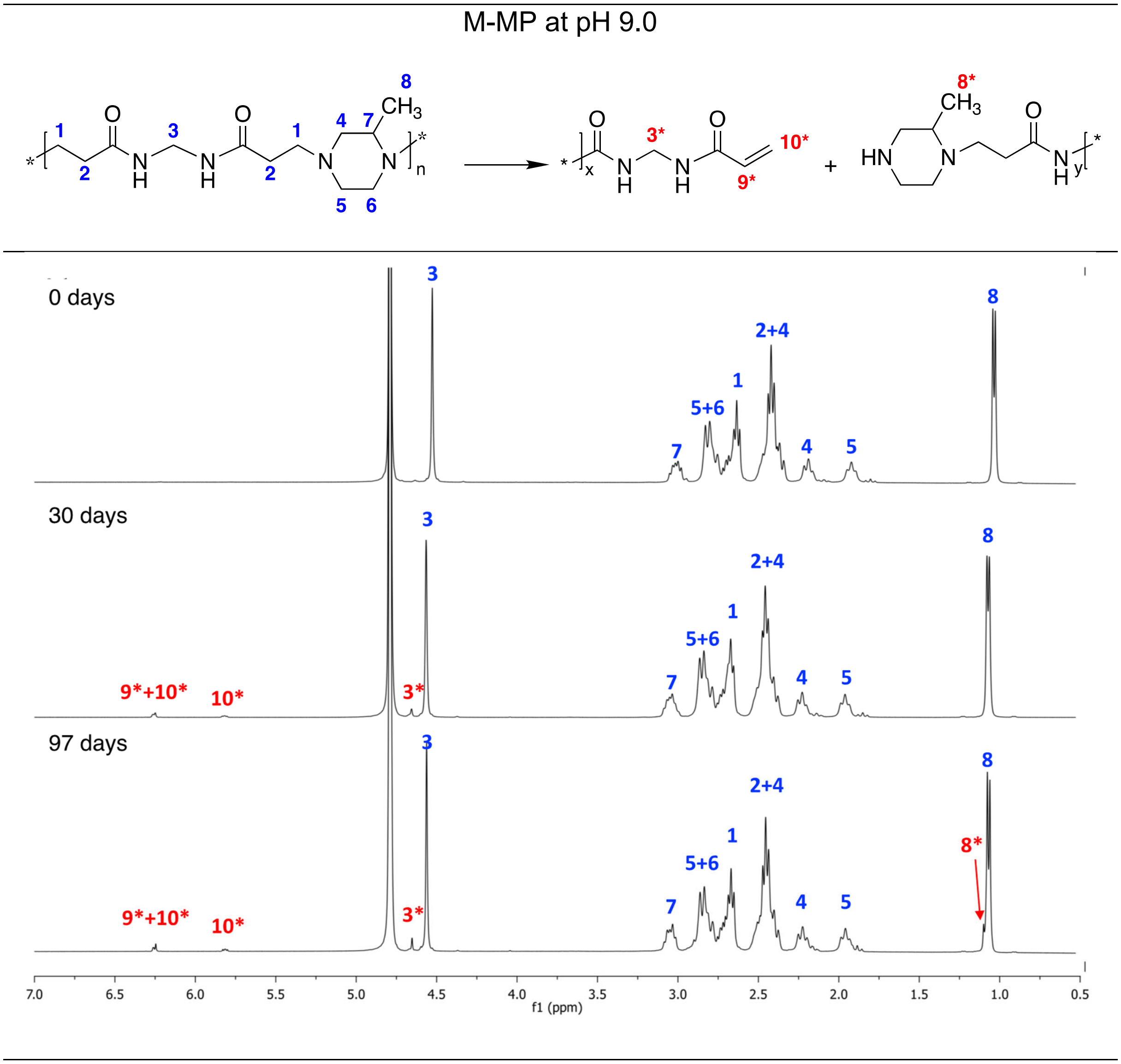

Figure S21. ^{1}H-NMR of M-MP at pH 9.0.

Degradation percent calculated both as

$[H_{3^*}/(H_3+H_{3^*})] \times 100$ and

$\{[(H_{9^*}+H_{10^*})/3]/H_7\} \times 100$

where H_n stands for the integral of the resonance peak of the hydrogen indicated in the subscript.

B-EA at pH 9.0

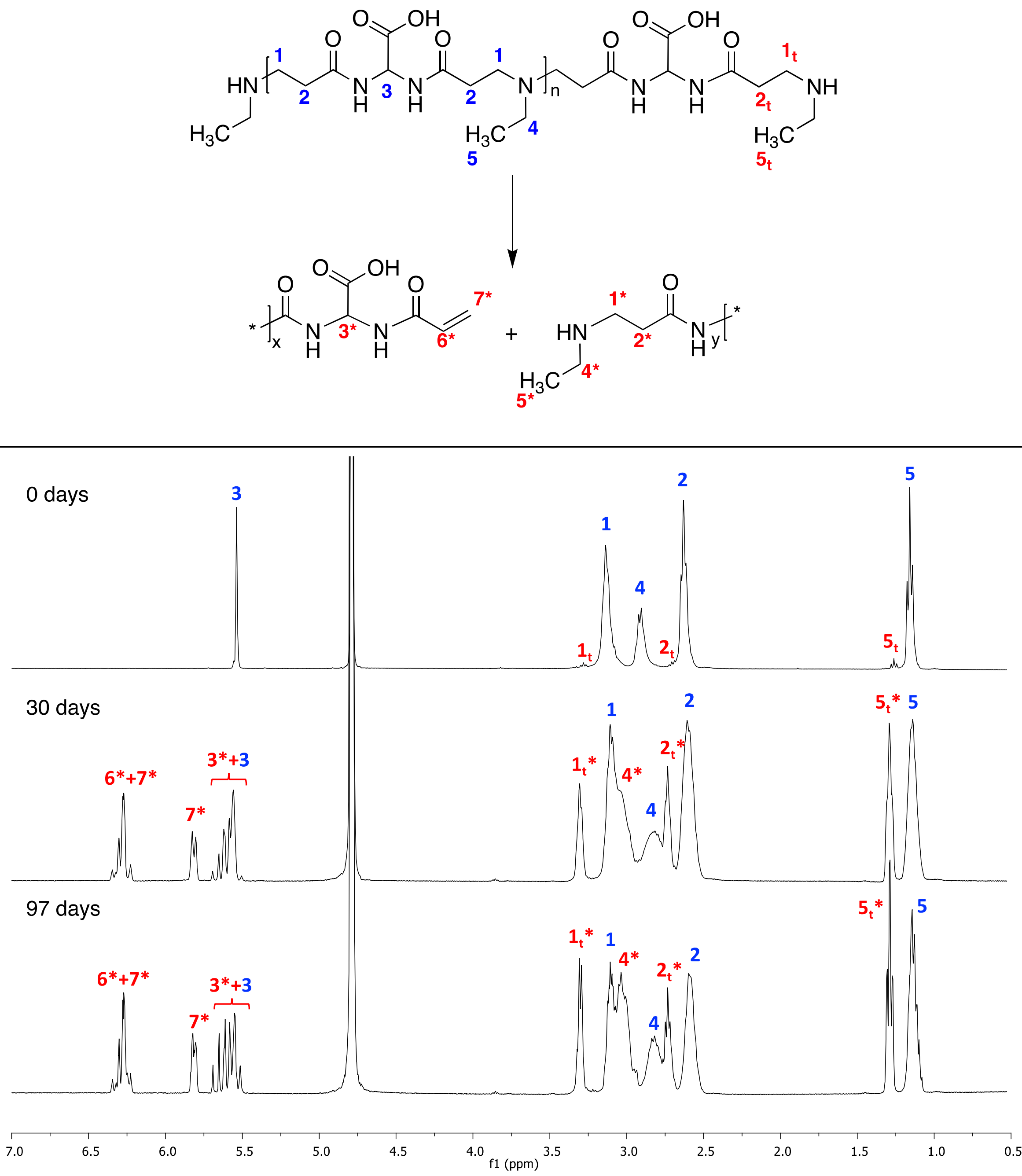

Figure S22. ^{1}H-NMR of B-EA at pH 9.0.

Degradation percent calculated both as

$[H_{5^*}/(H_5+H_{5^*})] \times 100$ and

$\{[(H_{6^*}+H_{7^*})/3/(H_3+H_{3^*})]\} \times 100$

where H_n stands for the integral of the resonance peak of the hydrogen indicated in the subscript.

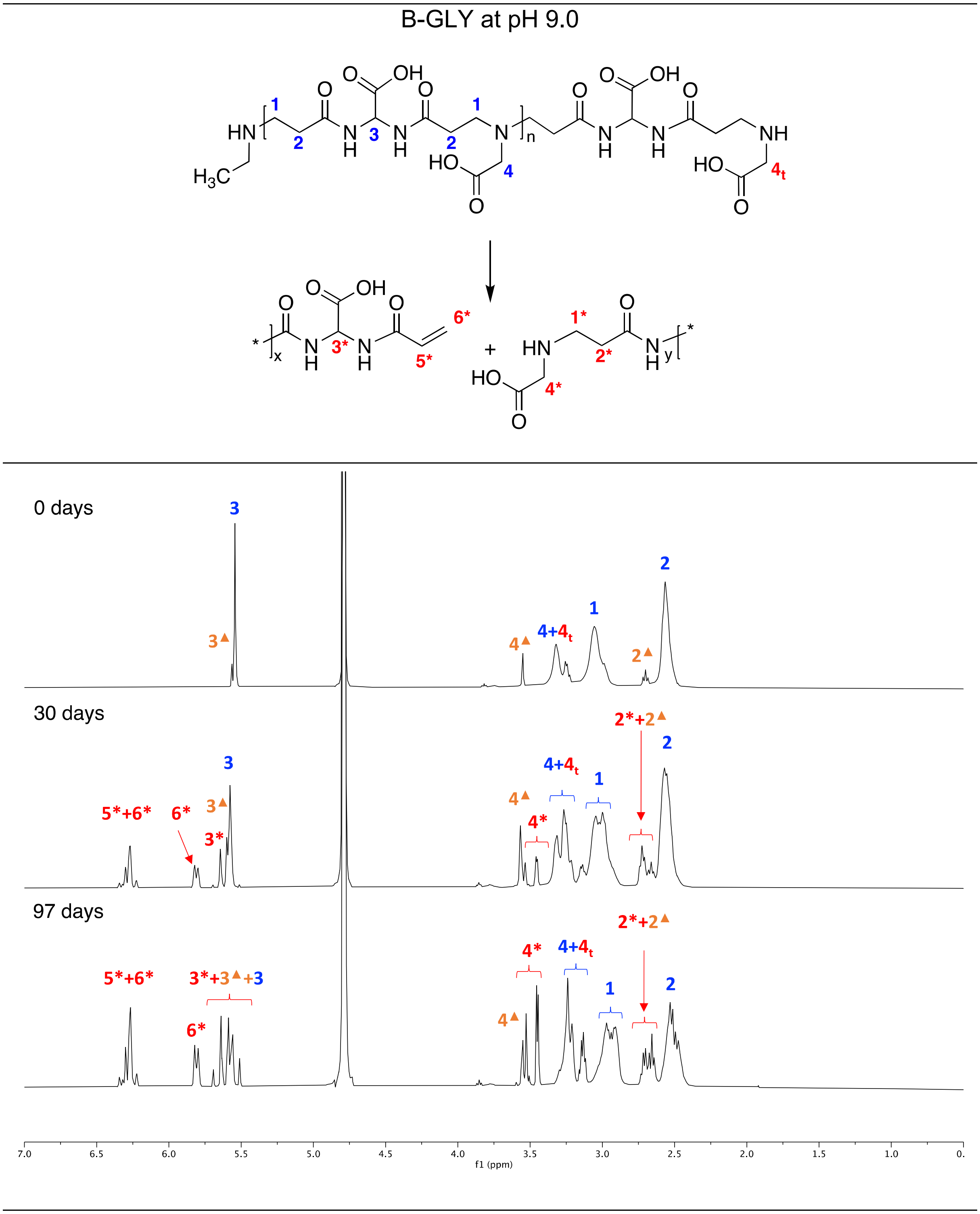

Figure S23. ^{1}H-NMR of B-GLY at pH 9.0.

▲ Side products with glycine terminals.

Degradation percent calculated as

$\{[(H_{5^*}+H_{6^*})/3/(H_3+H_{3^*})]\} \times 100$

where H_n stands for the integral of the resonance peak of the hydrogen indicated in the subscript.

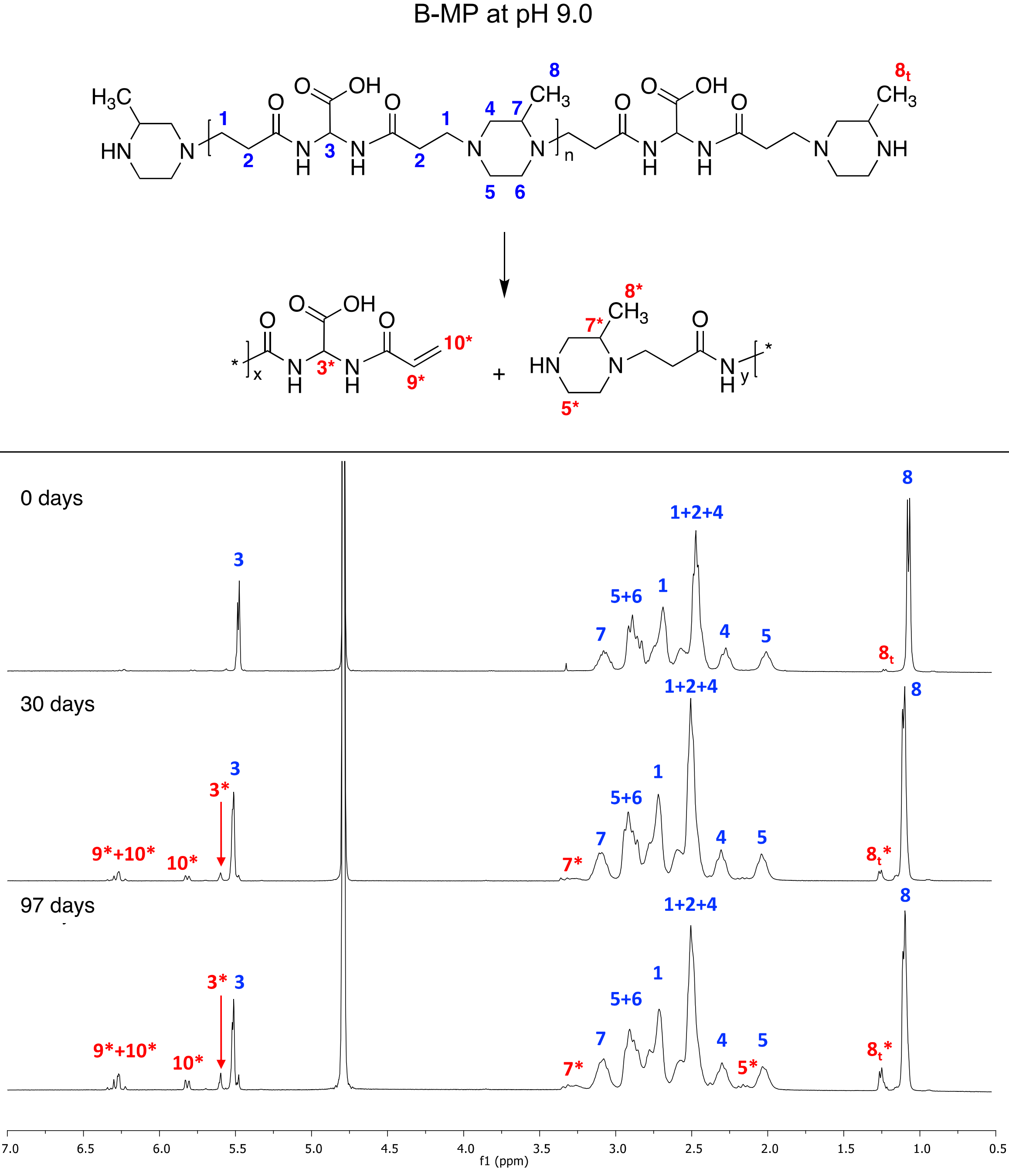

Figure S24. ^{1}H-NMR of B-MP at pH 9.0.

Degradation percent calculated both as

$[H_{3^*}/(H_3+H_{3^*})] \times 100$ and

$[H_{8^*}/(H_8+H_{8^*})] \times 100$ and

$\{[(H_{9^*}+H_{10^*})/3/(H_7+H_{7^*})]\} \times 100$

where H_n stands for the integral of the resonance peak of the hydrogen indicated in the subscript.

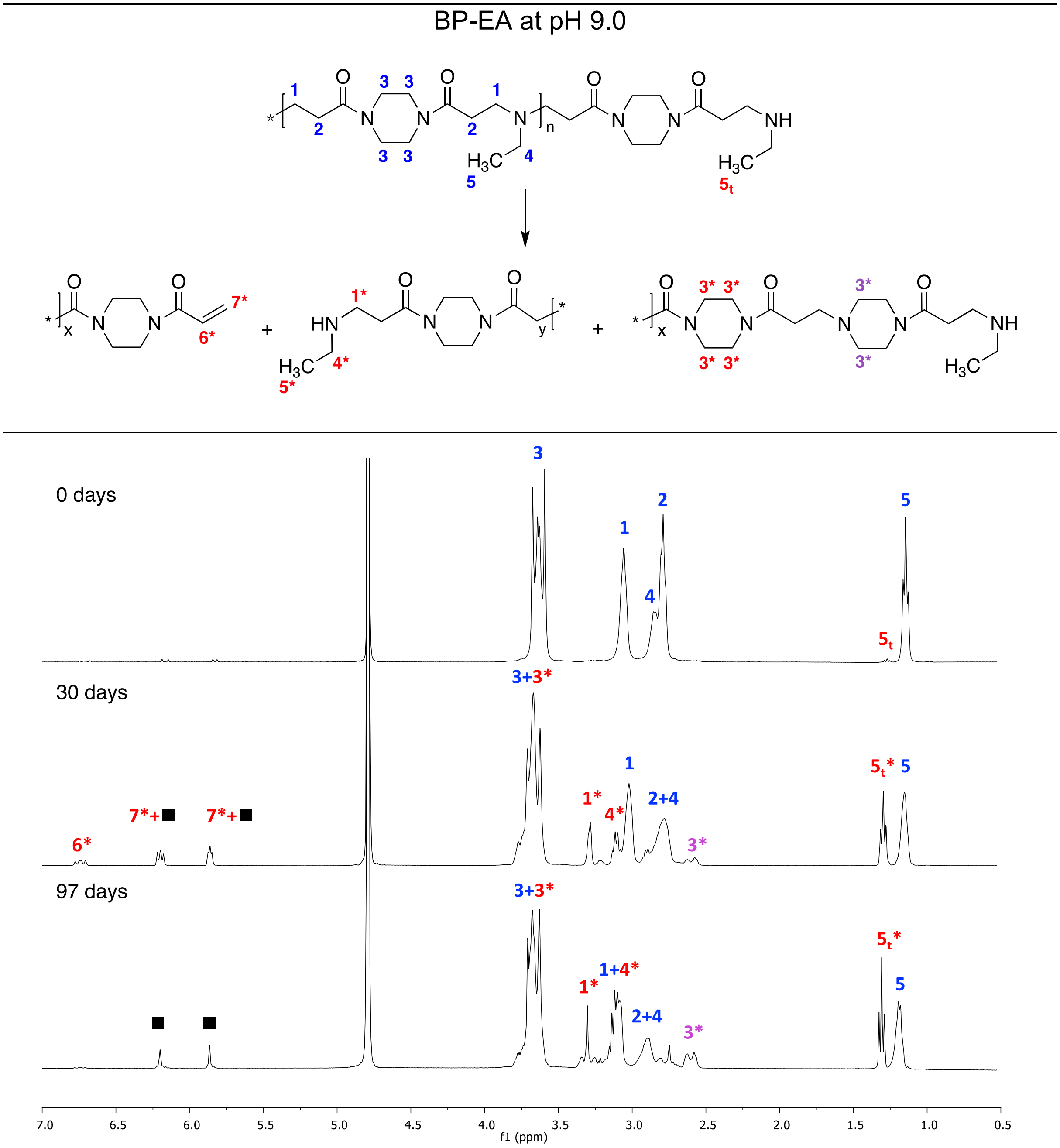

Figure S25. ^{1}H-NMR of BP-EA at pH 9.0.

■ Side products.

Degradation percent calculated both as

$[H_{5^*}/(H_5+H_{5^*})]\ x\ 100$ and

$\{[(H_{6^*}+H_{7^*})/3]/[(H_3+H_{3^*})/8]\}\ x\ 100$

where H_n stands for the integral of the resonance peak of the hydrogen indicated in the subscript.

BP-GLY at pH 9.0

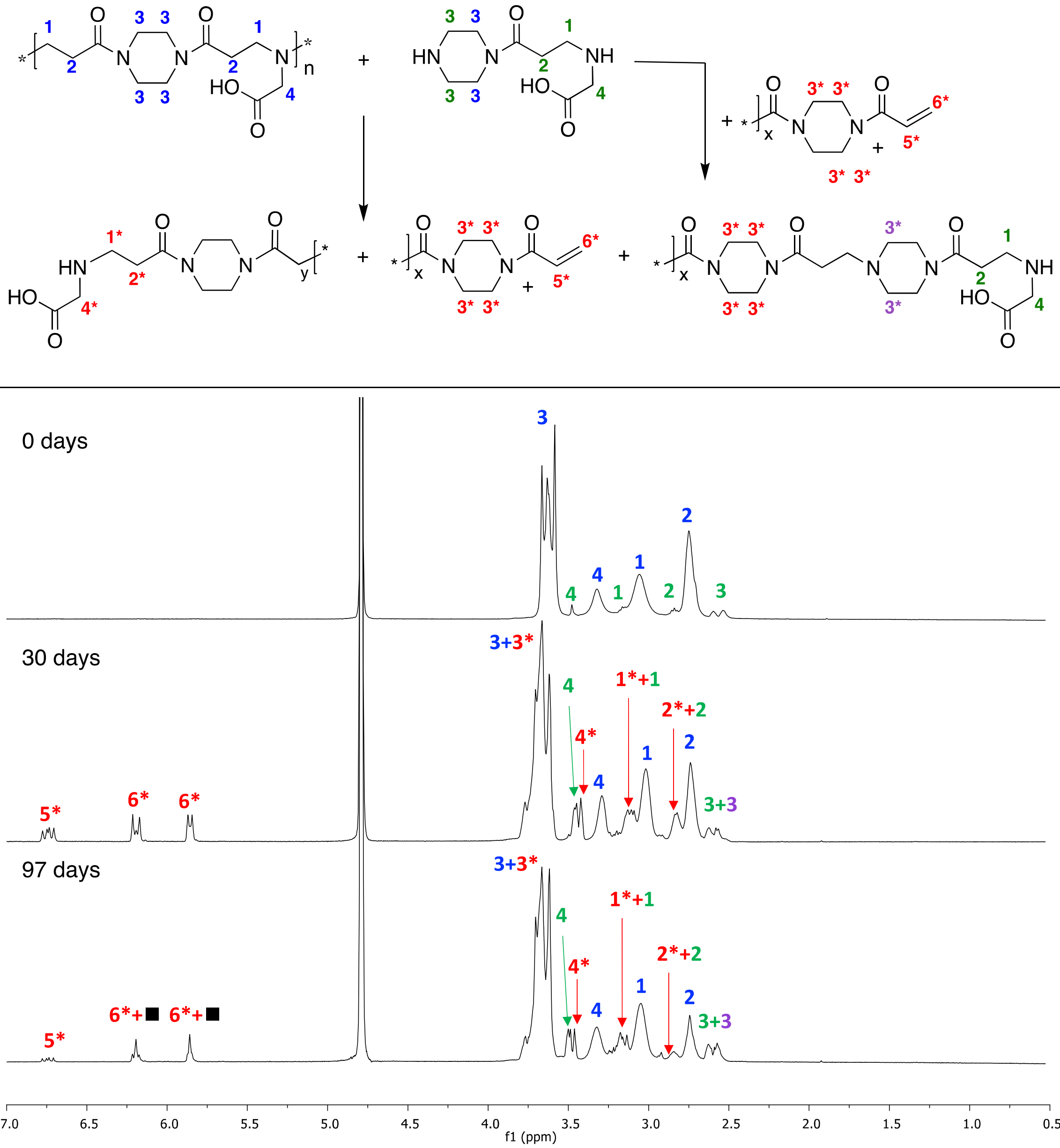

Figure S26. ^{1}H-NMR of BP-GLY at pH 9.0.

■ Side products.

Degradation percent calculated as

$\{[(H_{5^*}+H_{6^*})/3]/[(H_3+H_{3^*})/8]\} \times 100$

where H_n stands for the integral of the resonance peak of the hydrogen indicated in the subscript.

BP-MP at pH 9.0

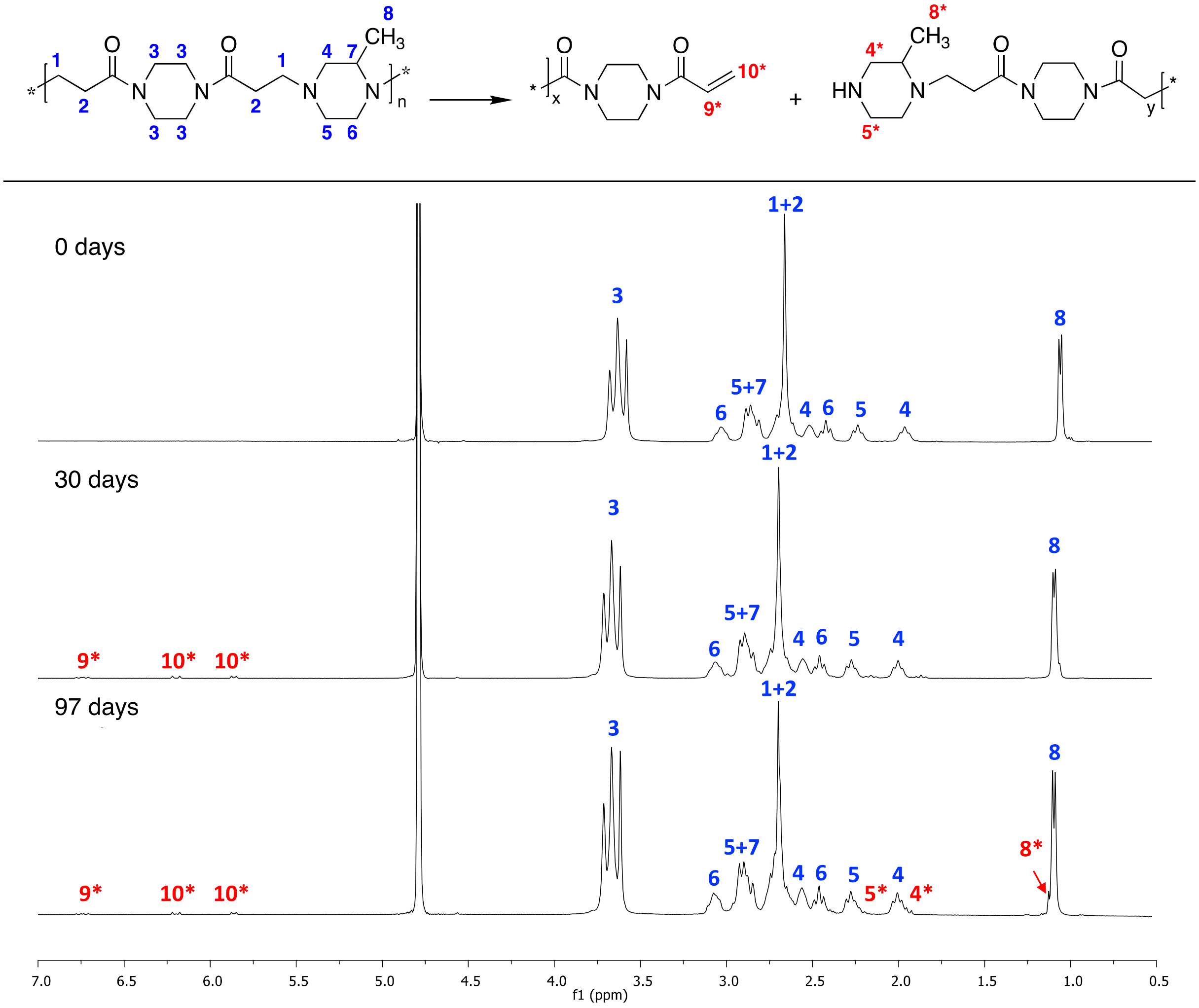

Figure S27. ^{1}H-NMR of BP-MP at pH 9.0.

Degradation percent calculated both as

$\{[(H_{9^*}+H_{10^*})]/[(H_8+H_{8^*})]\} \times 100$ and

$\{[(H_{9^*}+H_{10^*})/3]/[(H_3+H_{3^*})/8]\} \times 100$

where H_n stands for the integral of the resonance peak of the hydrogen indicated in the subscript.

M-GLY at pH 9.0 and 50 °C

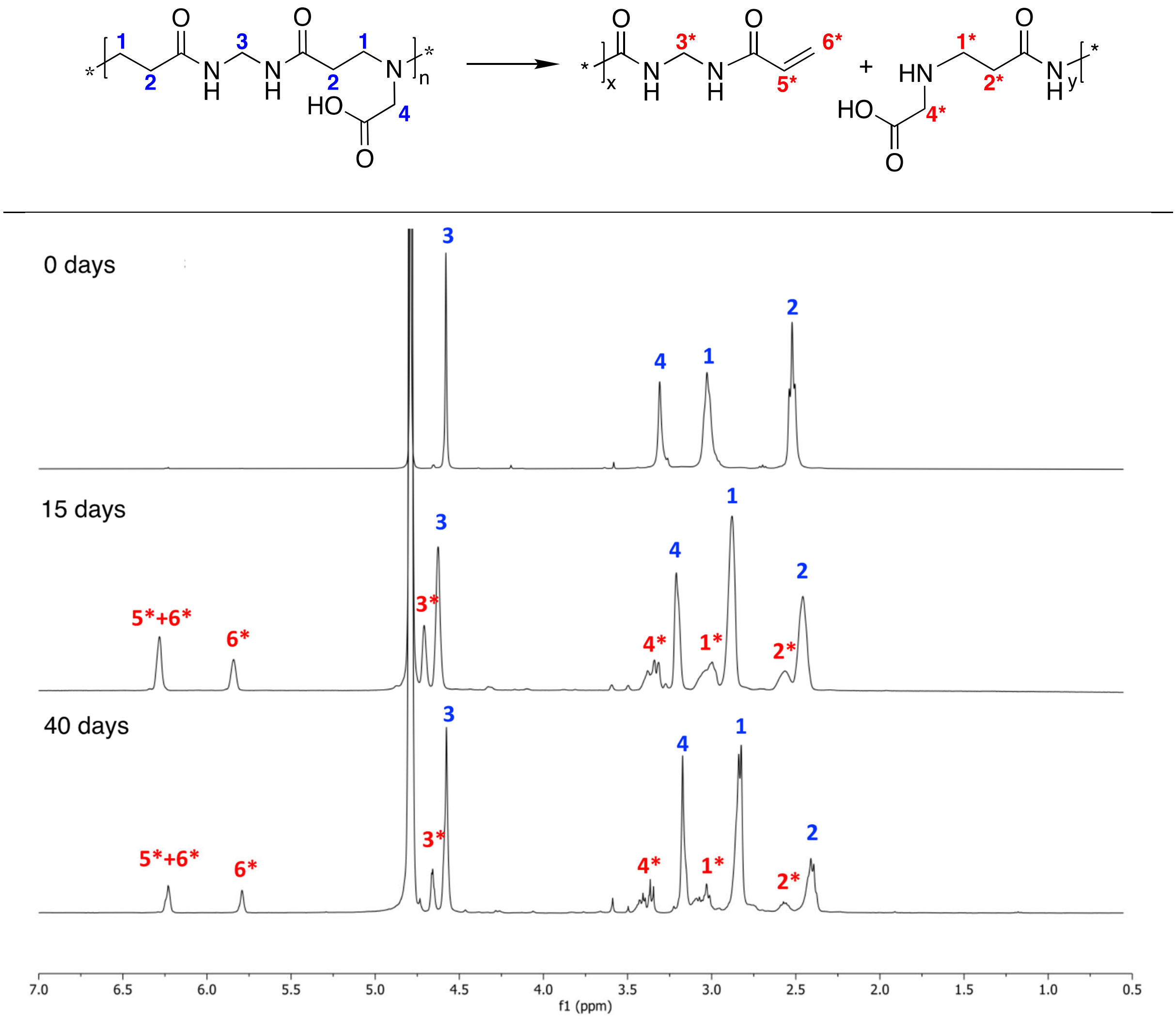

Figure S28. ^{1}H-NMR of M-GLY at pH 9.0 and 50 °C.

Degradation percent calculated both as

$[H_{3*}/(H_3+H_{3*})]\ x\ 100$ and

$\{[(H_{5*}+H_{6*})/3]/[(H_2+2H_{2*})/4]\}\ x\ 100$ and

$[H_{1*}/(H_1+2H_{1*})]\ x\ 100$

where H_n stands for the integral of the resonance peak of the hydrogen indicated in the subscript.
